# Virtual Home Care for Patients With Acute Illness

**DOI:** 10.1001/jamanetworkopen.2024.47352

**Published:** 2024-11-26

**Authors:** Josh Banerjee, Christopher Lynch, Hugh Gordon, Charles E. Coffey, Catherine P. Canamar, Soodtida Tangpraphaphorn, Karla Gonzalez, Neha Mahajan, Jan Shoenberger, Michael Menchine, Andrew Oh, Emily Johnson, Molly Grassini, Rachel Baden, Paul Holtom, Douglass Hutcheon, Brandon M. Wiley, Kusha Davar, Sheila Mallet-Smith, Margaret Sanfratello, Brenda Gallardo, Meixine Song, Nikole Swain, Maria Lydia Solis, Jenny Silva, Charmaine Pablico, Eduardo Aceves, Erica Bonilla, Ria Ashley Legaspi, Deisy M. Guevara, Karissa Lee, Christina Martinez, Michelle Banh, Dana Russell, Lissette Cervantes, Jacqueline Cervantes, Cesar Gonzalez, Phillip Sheth, Shadi Dowlatshahi, Alex Rosenberg, Pruthul Patel, Wei-An Lee, Tanzim Khan, Tze-Woei Tan, Michael Fong, Samuel S. Gordon, Brandi Clark, Victor Pena, Steven Dohi, Beatrisa Bannister, Roman Villalta, Priya Induru, Pauline Vuong, David Lwe, Karen Stoffel, Sam Oh, Christian Voyageur, Andrew Cool, Yong Lee, Stephen Lenh, Janet Luong, Gary Hanna, Jason N. Doctor, Bryan Munoz, Concepcion Castro, Edgar Solis, Nancy Blake, Roza Sakzalyan, Christopher J. Rodriguez, Christina Ghaly, Jorge Orozco, Hal F. Yee, Brad Spellberg

**Affiliations:** 1 Hospital Administration, Los Angeles General Medical Center, Los Angeles, California; 2 Urgent Care, Los Angeles General Medical Center, Los Angeles, California; 3 Hospital Medicine, Los Angeles General Medical Center, Los Angeles, California; 4 Quality, Los Angeles General Medical Center, Los Angeles, California; 5 Emergency Medicine, Los Angeles General Medical Center, Los Angeles, California; 6 Medicine, Los Angeles General Medical Center, Los Angeles, California; 7 Infectious Diseases, Los Angeles General Medical Center, Los Angeles, California; 8 Cardiology, Los Angeles General Medical Center, Los Angeles, California; 9 Department of Nursing, Los Angeles General Medical Center, Los Angeles, California; 10 Department of Pharmacy, Los Angeles General Medical Center, Los Angeles, California; 11 Endocrinology, Los Angeles General Medical Center, Los Angeles, California; 12 Limb Salvage Program, Rancho Los Amigos Medical Center, Downey, California; 13 Division of Cardiovascular Medicine, Keck School of Medicine of USC, Los Angeles, California; 14 Information Systems, Los Angeles General Medical Center, Los Angeles, California; 15 USC Price School of Public Policy, Los Angeles, California; 16 Public Information, Los Angeles General Medical Center, Los Angeles, California; 17 Los Angeles County Department of Health Services, Los Angeles, California

## Abstract

**Importance:**

Recent evolutions in clinical care and remote monitoring suggest that some acute illnesses no longer require intravenous therapy and inpatient hospitalization.

**Objective:**

To describe outcomes of patients receiving care in a new, outpatient, virtual, home-based acute care model called Safer@Home.

**Design, Setting, and Participants:**

This retrospective cohort analysis, conducted from September 1, 2022, through August 31, 2023, included 2466 patients treated at a safety net hospital in Los Angeles County for 10 core illnesses and 24 other acute illnesses for which patients are commonly hospitalized.

**Exposure:**

Outpatient, home-based, acute care with virtual monitoring and clinic visits in lieu of inpatient or in-home care.

**Main Outcomes and Measures:**

The primary measure was hospital length of stay. Secondary measures included all-cause mortality, 30-day readmission, return urgent care visit rates, and return emergency department (ED) visit rates.

**Results:**

Safer@Home provided care to 876 patients (mean [SD] age, 54.0 [14.5] years; 541 men [61.8%]) during the study period, compared with a cohort of 1590 patients (mean [SD] age, 52.3 [19.6] years; 901 men [56.7%]) with matching diagnoses who received standard, hospital-based care. Safer@Home patients had significantly shorter mean (SD) lengths of inpatient stay than the comparison cohort (1.3 [2.0] vs 5.3 [10.4] days; *P* < .001), totaling 3505 bed-days avoided (mean [SD], 4.0 [10.6] bed-days saved per patient), with no significant difference in all-cause mortality at last follow-up (2.6% [23 of 876] vs 4.0% [64 of 1590]; *P* = .07). Safer@Home patients and control patients also had no significant difference in the proportion experiencing 30-day hospital readmission (19.9% [174 of 876] vs 16.7% [266 of 1590]; *P* = .06). As intended, more Safer@Home than control patients had at least one 30-day return urgent care visit (37.3% [327 of 876] vs 5.2% [82 of 1590]; *P* < .001). In contrast, the Safer@Home and control cohorts did not significantly differ in experiencing at least one 30-day return ED visit (15.2% [133 of 876] vs 12.5% [199 of 1590]; *P* = .06). Safer@Home patients had significantly fewer mean (SD) total 30-day return ED visits per patient than control patients (0.19 [0.50] vs 0.21 [0.85]; *P* < .001).

**Conclusions and Relevance:**

In this cohort study, patients receiving acute, virtual, home care with remote monitoring and as-needed return urgent care visits had markedly shorter hospital stays than patients receiving standard inpatient hospital care, with no significant increase in mortality, ED revisits, or return hospitalizations. This new care model is promising for systems that cannot staff Medicare-compliant hospital-at-home visits.

## Key Points

**Question:**

Can hospital-like acute care be provided by an outpatient, virtual home-based model?

**Findings:**

In this cohort study, 876 patients receiving outpatient, virtual, home-based acute care spent 4 fewer days in the hospital than 1590 matched patients receiving standard hospital care, with no difference in 30-day readmission or mortality rates, and zero out-of-hospital deaths.

**Meaning:**

This study suggests that outpatient, virtual, home-based acute care, consisting of oral or inhalational therapy in lieu of intravenous therapy, remote monitoring, and as-needed return outpatient urgent care visits, is safe for a variety of acute illnesses and can be a useful model for systems that cannot staff in-home visits.

## Teaser

This cohort study compares outcomes of patients receiving care in an outpatient, virtual, home-based acute care model with outcomes of patients receiving standard hospital care.

## Introduction

Acute hospital care at home (AHCaH) has demonstrated promise nationally, with low rates of 30-day mortality and hospital readmission.^1^ However, Medicare standards for operating AHCaH programs require face-to-face evaluations, including vital signs measurement, performed 3 times daily in the patient’s home by hospital staff.^2,3^ Therefore, such programs require additional nursing beyond that necessary to staff hospital beds and can require substantial transit time if patients’ homes are widely dispersed and/or in areas with heavy traffic. For these reasons, the Los Angeles County Department of Health Services (LAC DHS), which operates the second-largest metropolitan health system in the US, has been unable to implement AHCaH programs.

However, in 2020, the LAC DHS developed an entirely virtual, acute, home care model for patients with COVID-19 pneumonia requiring oxygen. The program was found to be safe and effective^4^ and ultimately saved the LAC DHS from implementing crisis care during the 2020 winter COVID-19 surge. The program also demonstrated that some acute care, historically assumed to necessitate inpatient stay (ie, acute hypoxic respiratory failure requiring oxygen), can be safely delivered at home, with careful clinical selection criteria and ongoing virtual evaluation and management.

The LAC DHS therefore expanded this model for a variety of acute conditions for which patients usually require hospitalization. The expanded program, Safer@Home, was launched at the largest medical center in the LAC DHS, the Los Angeles General Medical Center (LA General), on September 1, 2022. Here we describe the program and report that patients treated through the Safer@Home program experienced considerable reductions in length of inpatient hospital stay, with excellent safety outcomes.

## Methods

### Study Design, Setting, and Population

LA General is a county-operated, public, 676-bed, level I trauma, safety net teaching hospital near downtown Los Angeles and is 1 of 3 public acute care hospitals in the LAC DHS system. We evaluated the outcomes of patients participating in the Safer@Home program in its first year, from its launch on September 1, 2022, through August 31, 2023. Reporting of this retrospective cohort study analysis follows the Strengthening the Reporting of Observational Studies in Epidemiology (STROBE) reporting guideline. The University of Southern California institutional review board approved this study as expedited and with a waiver of informed consent because data were collected for standard operations, rather than for study purposes, and were evaluated in a deidentified manner retrospectively.

### Safer@Home Care Model

The Safer@Home model was inspired by recent, extensive evidence that intravenous medications are not necessarily more effective than oral medications for numerous acute diseases that have historically required hospitalization^5,6,7,8,9,10^ and by the demonstration that acute, nasal canula oxygen could be safely managed at home rather than in the hospital.^4^ Instead of keeping such patients hospitalized, the Safer@Home program discharges eligible patients to home with entirely virtual clinical and vital signs monitoring, oral or inhalational therapy (no intravenous therapy used), and return urgent care visits when in-person evaluations are needed. Because Safer@Home does not involve any in-home visits by hospital personnel, the program does not meet Medicare reimbursement requirements for hospital-at-home.

The LA General Safer@Home team identified 10 core acute care diagnoses potentially amenable to remote treatment and monitoring (Box). Patients of all ages were eligible for Safer@Home only if they would otherwise be admitted to or kept in the hospital. We enrolled patients from the inpatient setting to facilitate earlier discharge or from the emergency department (ED), observation unit, or urgent care clinic to avoid hospital admission entirely.

Box. **Safer@Home Program General Eligibility Criteria, Core Protocolized Conditions, General Interventions, and Home Monitoring Equipment**

**General Eligibility Criteria**

- Plan at time of referral is either admission to hospital or continued hospitalization for inpatient monitoring and/or intravenous medications.
- Patient is hemodynamically stable with improving clinical trajectory.
- Patient can tolerate and is expected to absorb oral medications (eg, no short gut syndrome, presence of venting gastrostomy tube, persistent vomiting).
- There are no other reasons for continued evaluation and management in an acute care setting.
- Patient and/or caregiver can reliably follow up remotely with Safer@Home team.
- Patient and/or caregiver can provide accurate telephone number and discharge address (eg, home, recuperative care, quarantine housing).
- Patient and/or caregiver can demonstrate understanding of discharge instructions.
- Patient can reliably and rapidly return to medical facility if deemed necessary by clinician.
- All ages are eligible.

**Core Protocolized Conditions**

- Cellulitis
- Diabetic foot infection (with or without osteomyelitis)
- Nonspinal osteomyelitis
- Spinal osteomyelitis
- Pyelonephritis or complicated urinary tract infection
- COVID-19 pneumonia
- Non–COVID-19/other viral pneumonia
- Bacterial pneumonia
- COPD and asthma exacerbation
- Heart failure exacerbation

**General Safer@Home Interventions**

- Nurse provides education, medications, and equipment to patient prior to discharge; monitoring happens in real time.
- Nurse calls 12-18 h after discharge, then at least daily, 7 d a week until patient completes program.
- Physician reviews case daily and provides decision support and direct teleevaluation when indicated.
- Nurse and/or physician determine if patient needs to return for evaluation at facility.
- Expedited return trips to facility for laboratory tests, imaging, medications, or clinician visits are coordinated by nurses with roundtrip transportation.

**Durable Medical Equipment for Home Monitoring, Dispensed by Diagnoses**

- Cellulitis, diabetic foot infection, nonspinal osteomyelitis: thermometer, pulse oximeter
- Pyelonephritis, complicated urinary tract infection: thermometer, pulse oximeter
- COPD or asthma exacerbation: thermometer, pulse oximeter, oxygen tank, concentrator, nebulizer
- Bacterial COVID-19 or other viral pneumonia: thermometer, pulse oximeter, oxygen tank, concentrator
- Heart failure exacerbation: scale, pulse oximeter, thermometer, blood pressure device

Abbreviation: COPD, chronic obstructive pulmonary disease.

### Safer@Home Eligibility Criteria and Treatment Plans

Patients eligible for Safer@Home met both prespecified general eligibility criteria (Box) and additional criteria specific to each eligible diagnosis (eBox 1 in Supplement 1). After program entry, all Safer@Home patients received education, discharge medications, and disease-specific durable medical equipment prior to discharge (Box). Oral or inhalational medication treatment plans, specifying drug, dose, route, and duration, were followed via disease-specific protocols (eBox 2 in Supplement 1).

**Figure 1.**
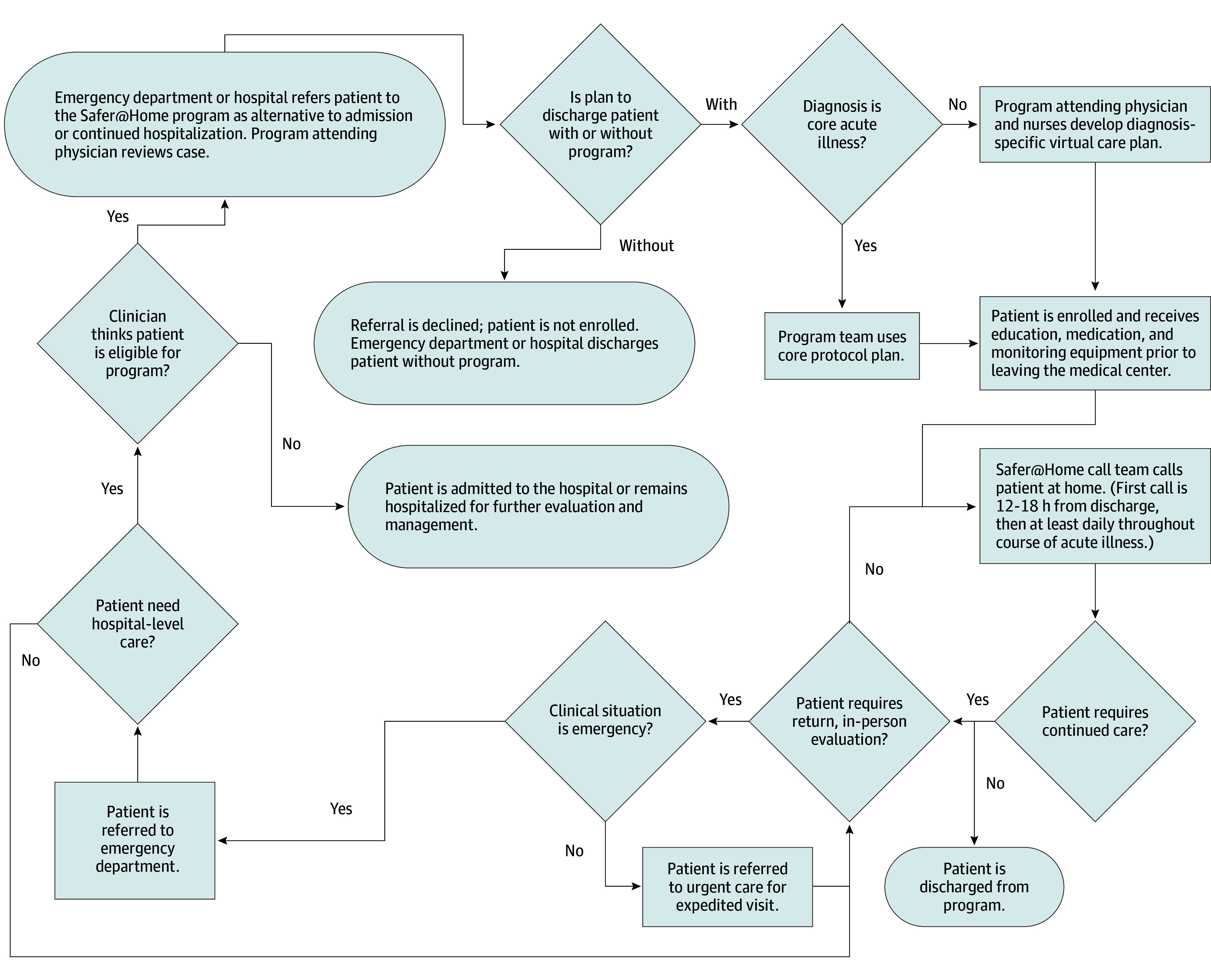
Safer@Home Patient Enrollment, Follow-Up, and Care Escalation Workflow

Within 12 to 18 hours of leaving the medical center, all patients received their first call from a Safer@Home nurse (Figure 1). The nurse used a script (eTable 1 in Supplement 1) based on symptoms and vital signs to evaluate the patient and determine whether they could continue virtual home evaluation and management with routine daily briefing to the Safer@Home attending physician, whether real-time consultation with the Safer@Home attending physician was needed, or whether the patient needed immediate direction to either urgent care or the ED for care escalation.

Safer@Home nurses and hospitalists provided coverage 12 hours a day, 7 days a week. If, after daily review of each patient, the hospitalist recommended further diagnostics, medication changes, or face-to-face evaluation, the nurses worked with the patient, pharmacy, laboratory, urgent care clinic, and transportation to coordinate delivery or expedited return to the medical center for that service. The team followed up with the patient until the nurses and attending physician agreed the patient no longer required further virtual care, at which point the patient was discharged from the Safer@Home program. The Safer@Home medical team documented all orders in the electronic health record (EHR).

### Data Collection

We retrospectively extracted demographic data, including age, gender identity, and race and ethnicity (determined by the patient during intake: Asian, Black, Hispanic, White, or other [a category reported by the patient]), from the Vizient Clinical Data Base. Race and ethnicity were included to ensure the patients were matched. We also retrospectively extracted clinical data from the Vizient Clinical Data Base, including case mix index, defined as the mean of the relative weights of the Medicare Severity Diagnostic Related Groups (MS DRGs) assigned to patients at discharge, and expected mortality, calculated by the Vizient Clinical Data Base using proprietary algorithms, based on age and the total sum of *International Statistical Classification of Diseases and Related Health Problems, Tenth Revision, Clinical Modification* and *Current Procedural Terminology* codes captured for the patients.

### Safer@Home and Comparison Cohorts

To identify other hospitalized patients with diseases similar to those of the patients enrolled in the Safer@Home program, we began by creating listings of principal and secondary diagnoses, procedure codes, and DRGs coded for all patients cared for via Safer@Home. After confirming that the coding algorithms identified all Safer@Home patients treated with each diagnosis, we used the same algorithms to run against all admissions to the hospital during the 12-month study period, excluding the Safer@Home patients, to identify the initial pool of all potential control patients.

**Figure 2.**
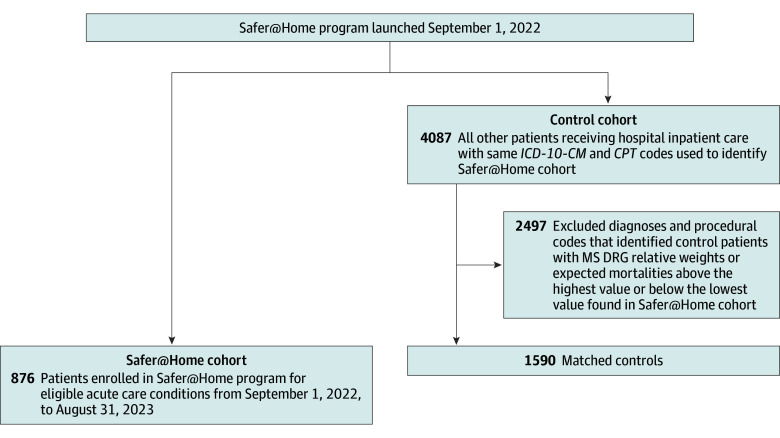
Safer@Home and Hospitalized Patient Cohorts *CPT* indicates *Current Procedural Terminology*; *ICD-10-CM*, *International Statistical Classification of Diseases and Related Health Problems, Tenth Revision, Clinical Modification*; and MS DRG, Medicare Severity Diagnostic Related Group.

To refine matching of the control pool for each diagnosis, the coding algorithms were then iteratively adjusted by adding exclusionary diagnoses or procedure codes uniquely found for potential control patients who had DRG relative weights (used to calculate case mix index) or expected mortalities above the highest value or below the lowest value seen for the Safer@Home patients. Iterative cycles continued until final diagnostic algorithms were established that enabled matching of Safer@Home patients and control patients on case mix index and expected mortality and subsequently based on gender identity and race and ethnicity (Figure 2; eTable 2 in Supplement 1).

Because DRGs are normally assigned only to hospital inpatients, DRGs were imputed by the study team for patients entering the Safer@Home program from urgent care or the ED. Specifically, the same MS DRGs or All Patient Related (APR) DRGs applied to typical inpatients with a specific diagnosis (eg, MS DRG 603 and APR DRG 383 for cellulitis) were also applied to patients with those diagnoses entering the program from the ED or urgent care.

### Patient Outcomes Analyses

The primary outcome was mean length of hospital stay for patients treated via the Safer@Home program vs controls. To be conservative, lengths of stay for Safer@Home patients enrolled from the ED or observation were counted as inpatient lengths of stay by comparing arrival times to the ED with departure times from ED or observation. In contrast, all control patients were admitted to the hospital, and their lengths of stay in the ED or observation prior to admission were not included.

As an additional sensitivity analysis, we compared the observed lengths of stay for Safer@Home patients and control inpatients with their expected lengths of stay based on their assigned APR DRGs. Expected lengths of stay of APR DRGs^11^ were used in lieu of other metrics because APR DRGs result in a more accurate correlation with hospital lengths of stay and resource utilization than other measures, including MS DRGs.^12,13,14,15^

Finally, as additional sensitivity analyses, we compared the mean (SD) length of stay for Safer@Home patients with several other control cohorts: (1) controls from LA General during the year prior to program implementation (September 1, 2021, to August 31, 2022) and (2) concurrent controls from the other 2 acute care hospitals in our LAC DHS system, Harbor-UCLA Medical Center and Olive View-UCLA Medical Center, which care for the same population of underserved patients in our public hospital system.

Safety and balancing measures included all-cause mortality at last follow-up, 30-day mortality, 30-day hospital readmission, 30-day return ED visits, and 30-day return urgent care visits. We calculated the interval measures for follow-up visits as the time of patient discharge from either index ED or hospital encounter to the time of arrival at subsequent encounter.

For the 3 highest frequency Safer@Home diagnoses, we compared the proportion of patients presenting to the ED who were admitted to the hospital during the study period vs the baseline year at LA General (ED case definitions in eTable 3 in Supplement 1). We conducted the same comparison analyses for control patients at Harbor-UCLA and Olive View-UCLA Medical Centers.

### Statistical Analysis

Results from the Kolmogorov-Smirnov test of normality for continuous data (eg, hospital length of stay) directed us to analyze group comparisons with the *t* test for normally distributed data or the Mann-Whitney test for nonnormally distributed data. Sensitivity analyses of concurrent control cohorts were conducted pairwise by using the Tukey-Kramer test. Cohort comparisons of proportional data (eg, dichotomized length of stay, mortality, and return hospitalization data) used the χ^2^ test or the Fischer exact test for sample sizes of 5 or fewer.

We performed multiple regression to assess whether measured confounders were associated with the primary outcome, observed length of stay. To address potential unmeasured confounding, we also calculated an E-value, according to methods described by VanderWeele and Ding.^16^ We used a dichotomized relative risk calculation for the E-value because we found minimal literature on the calculation of an E-value for continuous data. All statistical testing was 2-sided, α = .05. Data were analyzed using KyPlot, version 5.0 (KyensLab Inc) or Stata, version 17.0 (StataCorp LLC).

## Results

### Safer@Home and Control Cohort Populations

A total of 876 Safer@Home patients (mean [SD] age, 54.0 (14.5) years; 541 men [61.8%] and 335 women [38.2%]) received treatment during the 12-month study period (mean [SD], 2.4 [1.8] patients per day) (Table 1). Of these patients, 484 (55.3%) entered the program from the ED (404 [46.1%]), observation area (67 [7.6%]), or urgent care (13 [1.5%]), and 392 (44.7%) came from the inpatient setting. Of the Safer@Home patients, 823 (94.0%) received treatment for 1 of the 10 core protocolized conditions, while 53 (6.1%) had 1 of 24 other ad hoc diagnoses (eTables 4-15 in Supplement 1).

**Table 1.** Patient and Clinician Characteristics

Characteristic	Safer@Home cohort (n = 876)	Comparison cohort (n = 1590)	*P* value
Care setting at discharge, No. (%)			
Hospital (inpatient)	392 (44.7)	1590 (100)	NA
Emergency department (outpatient)	404 (46.1)	0	
Observation (outpatient)	67 (7.6)	0	
Urgent care (outpatient)	13 (1.5)	0	
Patient age, mean (SD), y	54.0 (14.5)	52.3 (19.6)	.15
Patient gender identity, No. (%)			
Female	335 (38.2)	688 (43.3)	.14
Male	541 (61.8)	901 (56.7)	
Patient race and ethnicity, No. (%)^a^			
Asian	19 (4.9)	75 (4.7)	.07
Black	14 (3.6)	108 (6.8)	
Hispanic	326 (83.2)	1235 (77.7)	
White	10 (2.6)	59 (3.7)	
Other^b^	26 (6.6)	113 (7.1)	
Patient case mix index, mean (SD)	1.3 (0.7)	1.3 (0.6)	.72
Patient expected mortality, mean (SD)	0.02 (0.5)	0.02 (0.06)	.45

Abbreviation: NA, not applicable.

^a^ Race and ethnicity data were only available for Safer@Home patients enrolled from the inpatient setting and not from those enrolled from the emergency department, observation, or urgent care clinic.

^b^ Based on patient preference as entered by the nurse in the electronic medical record; “other” was a category self-reported by the patient.

By comparison, 4087 patients with similar diagnoses received care as inpatients not in the Safer@Home program during the study period, of whom 2497 were excluded due to lack of matching on case mix index, expected mortality, gender identity, or race and ethnicity (Figure 2). After exclusions, we identified 1590 matched control inpatients during the study period (Table 1). Of these matched controls, 1354 patients had 1 of the 10 core protocolized conditions, and 236 had ad hoc conditions (eTables 4-15 in Supplement 1). Across all diagnoses, data on age, gender identity, race and ethnicity, case mix index, and expected mortality were similar between the Safer@Home and comparison cohorts (eTables 4-15 in Supplement 1).

### Patient Outcomes

Safer@Home patients had a significantly shorter overall mean (SD) length of stay than matched concurrent control patients (1.3 [2.0] vs 5.3 [10.4] days; *P* < .001), resulting in a total of 3505 inpatient bed days avoided (with a mean [SD] of 4.0 [10.6] bed-days saved per patient) (Table 2). We conducted a multiple regression sensitivity analysis to evaluate the association of measured potential confounders with the length of stay outcome, which demonstrated that each of the included variables (Safer@Home vs control cohort, MS-DRG relative weight, and expected mortality) was significantly and independently associated with observed length of stay (*R*^2^ = 0.08; *P* < .001) (eTable 16 in Supplement 1). We also assessed the risk of unmeasured confounders being associated with length of stay between the cohorts. Safer@Home patients had a relative risk of 2.37 (95% CI, 2.16-2.60) for having a length of stay of 1 day or less compared with control patients, with an E-value for unmeasured confounding of 4.17 (Table 2).

**Table 2.** Patient and Program Outcomes

Outcome	Safer@Home cohort	Comparison cohort	*P* value
Hospital length of stay, mean (SD), d	1.3 (2.0)	5.3 (10.4)	<.001
Hospital length of stay ≤1 d			
Observed No./total No. (%)	584/876 (66.7)	447/1590 (28.1)	<.001
Relative risk (95% CI)^a^	2.37 (2.16-2.60)	NA	<.001
Patients with at least one 30-d urgent care visit, No. (%)	327 (37.3)	82 (5.2)	<.001
No. of 30-d urgent care visit per patient, mean (SD)^b^	0.61 (0.96)	0.08 (0.51)	<.001
Patients with at least one 30-d emergency department visit, No. (%)^b^	133 (15.2)	199 (12.5)	.06
No. of 30-d emergency department visits per patient, mean (SD)^b^	0.19 (0.50)	0.21 (0.85)	<.001
30-d Hospital readmission, No. (%)	174 (19.9)	266 (16.7)	.06
30-d Mortality, No. (%)	4 (0.5)	16 (1.0)	.13
Mortality at last follow-up, No. (%)	30 (3.4)	75 (4.7)	.13
Successful Safer@Home nurse calls, mean (SD)	3.7 (2.4)	NA	NA
Total Safer@Home nurse calls, mean (SD)	6.6 (4.9)	NA	NA
Days in Safer@Home program, mean (SD)	6.1 (3.2)	NA	NA
Follow-up, mean (SD), d^c^	267 (192)	231 (200)	NA

Abbreviation: NA, not applicable.

^a^ E-value of 4.17.

^b^ Urgent care and emergency department counts do not include urgent care or emergency department visits that converted into hospital admissions.

^c^ Date of last follow-up is last clinical encounter in electronic health record as of June 7, 2024.

As an additional sensitivity analysis, the expected mean (SD) length of stay of Safer@Home patients by their APR DRGs was similar to the control cohort length of stay, 5.4 (1.9) days. Patients with similar diagnoses cared for at LA General during the baseline year, which was prior to the program implementation, had a longer mean (SD) length of stay than Safer@Home patients, 5.0 (7.8) days, as did patients with matching diagnoses cared for concurrently during the study period at Harbor-UCLA and Olive-View UCLA Medical Centers (eTable 17 in Supplement 1).

During a mean (SD) of 6.1 (3.2) days of virtual follow-up, 327 Safer@Home patients (37.3%) required a total of 538 return visits (mean [SD], 0.61 [0.96] visits per patient) to urgent care, while only 82 control patients (5.2%) required a total of 132 return visits to urgent care visits (mean [SD], 0.08 [0.51] visits per patient; *P* < .001) (Table 2). By contrast, the proportion of Safer@Home and control patients with at least one 30-day return ED visit (133 [15.2%] vs 199 [12.5%]; *P* = .06) and hospital readmission (174 [19.9%] vs 266 [16.7%]; *P* = .06) were not significantly different. Patients in the Safer@Home cohort had a significantly lower mean (SD) rate of return ED visits per person than control patients during follow-up (0.19 [0.50] vs 0.21 [0.85]; *P* < .001).

For the Safer@Home and comparison cohorts, there was no significant difference between mortality rates at 30 days (4 [0.5%] vs 16 [1.0%]; *P* = .13) and last follow-up (30 [3.4%] vs 75 [4.7%]; *P* = .13) (Table 2). There were no out-of-hospital deaths for patients enrolled in the Safer@Home program. There was also no difference between the Safer@Home and comparison cohorts in all-cause mortality at last follow-up (2.6% [23 of 876] vs 4.0% [64 of 1590]; *P* = .07).

The 3 highest-frequency Safer@Home diagnoses were diabetic foot infections, pyelonephritis or complicated urinary tract infections, and cellulitis. The proportion of patients presenting to the ED with these diagnoses who were admitted to the hospital significantly decreased at LA General during the study period compared with the baseline year prior (eTable 18 in Supplement 1). In contrast, there were no significant differences in the ratios of patients admitted for these conditions at either Harbor-UCLA or Olive View-UCLA Medical Centers.

## Discussion

We found that a virtual, home-based, acute care model enabled substantial avoidance of inpatient hospital days. Mortality at last follow-up and 30-day return hospitalizations were not significantly different between patients in the Safer@Home program and matched control patients receiving inpatient hospital care. Safer@Home patients had fewer 30-day return ED visits than control patients. In contrast, Safer@Home patients demonstrated significantly higher return urgent care utilization. The higher urgent care utilization was a specifically intended mechanism of follow-up in the program, which sought to replace inpatient care with home care and with urgent care visits when remote monitoring of patient symptoms and vital signs warranted face-to-face evaluation.

This new virtual care model was enabled by recent changes in clinical practice (eg, the establishment by numerous randomized clinical trials that intravenous antibiotics are not more effective than oral antibiotics for many diseases but are less safe).^5,6,7,8^ There are also no data demonstrating that intravenous diuresis for heart failure or inpatient nasal canula oxygen was more effective than oral diuresis or home oxygen with close monitoring.^4^ Thus, the historically standard practice of admitting patients just for therapy and/or only to monitor patients to ensure clinical improvement is no longer clinically necessary in many cases, particularly given the recent availability of portable pulse oximeters and other means to monitor patients virtually.

### Limitations

Our study has several limitations. This is a hypothesis-generating observational study, prone to multiple forms of confounding. This study also did not include measures of patient satisfaction or functional status after hospital discharge, and it was conducted in a large, urban public hospital system, which may not extrapolate to other care settings. A randomized clinical trial could include prospective patient-reported outcome measures, could include more varied hospital settings, and would more definitively establish causality, if there is a determination of equipoise regarding safety of avoiding admission for such patients.

Although the Safer@Home program had numerically higher 30-day rehospitalization rates and numerically lower mortality rates at 30 days and last follow-up, the differences were not statistically significant. In the absence of the program, all Safer@Home patients would have remained in the hospital for a mean of 4 additional days; thus, any numerical, nonsignificant difference in rehospitalization was much smaller than the duration of inpatient stays that would have resulted in the absence of the program. Furthermore, the Safer@Home and control cohorts were similar by age, gender identity, race and ethnicity, case mix index (as a marker of resource utilization), expected mortality (as a marker of disease severity), and diagnoses and procedures. Multiple regression adjustment for measured confounders did not alter the results, and the E-value of 4.17 indicates that only a very strongly associated unmeasured confounder could have been associated with the results, providing assurance that lesser confounding effects were not associated with the length of stay outcome.

Another potential limitation is the risk that some enrolled patients would have been sent home even without the Safer@Home program. We aggressively attempted to exclude these patients by actively denying referrals unless they otherwise required hospitalization. Nearly half of the Safer@Home patients were discharged from the inpatient setting, confirming they were sick enough to require admission. Finally, we confirmed that patients presenting to the ED with the highest-volume target diagnoses were less frequently admitted to LA General during the study period, a phenomenon not observed at control safety net hospitals, providing evidence that the program did enable patients who otherwise would have been admitted to the hospital to be cared for at home.

One operational limitation was that less than half of potentially eligible patients entered the Safer@Home program during the study period, based on the ratio of matched Safer@Home and control patients, suggesting that there remains considerable room for programmatic expansion. Culture change from the traditional practice of medicine, in which most such patients are admitted to the hospital, was the biggest barrier we encountered and will likely be the biggest barrier to adoption in other health systems.

## Conclusions

We found that a novel, virtual, at-home alternative to hospitalization achieved a mean 4-day reduction in inpatient length of stay across numerous diagnoses, with a reduced 30-day return ED visits and with no significant change in mortality. The trade-off for substantially reducing inpatient stay was a 1 in 3 increase in the proportion of patients requiring a 30-day return outpatient visit to urgent care. The program was readily implemented in an urban, teaching, safety net hospital with a large service region and staffing constraints. This model could be a viable alternative to home-based programs that require deployment of staff to patients’ homes.

## Notes

Supplement 1. **eBox 1.** Condition-Specific Enrollment Criteria **eBox 2.** Condition-Specific Treatment Plans **eTable 1.** Safer@Home Nurse Call Script **eTable 2.** Diagnostic/Procedural Criteria for Identification of Control Cohorts of Hospitalized Patients **eTable 3.** Definition of Cases of Cellulitis, Diabetic Foot Infection, and Pyelonephritis Presenting to Emergency Department (ED), by ICD-10-CM Codes **eTable 4.** Characteristics of Patients With “Core” Diagnoses **eTable 5.** Characteristics of Patients With Other Diagnoses **eTable 6.** Characteristics of Patients With Cellulitis **eTable 7.** Characteristics of Patients With Diabetic Foot Infections **eTable 8.** Characteristics of Patients With Osteomyelitis **eTable 9.** Characteristics of Patients With Pyelonephritis **eTable 10.** Characteristics of Patients With Bacterial Pneumonia **eTable 11.** Characteristics of Patients With COPD Exacerbation **eTable 12.** Characteristics of Patients With CHF **eTable 13.** Characteristics of Patients With Asthma **eTable 14.** Characteristics of Patients With COVID Pneumonia **eTable 15.** Characteristics of Patients With Acute Hypoxic Respiratory Failure Due to Viral infection **eTable 16.** Multivariable Linear Regression Model of Predictors of Observed Hospital Length of Stay (Days) **eTable 17.** Sensitivity Analysis, Observed Lengths of Stay for Enrollment and Matched Non-Enrollment Cohorts **eTable 18.** Hospital Admission Rates From Emergency Department for the Three Most Frequently Enrolled Safer@Home Diagnoses

Supplement 2.
Data Sharing Statement
