# Supplementary material for: Virtual Home Care for Patients With Acute Illness
Source: JAMA Netw Open. 2024 Nov 26;7(11):e2447352. doi: 10.1001/jamanetworkopen.2024.47352 (PMC11600231; doi:10.1001/jamanetworkopen.2024.47352)
Supplement: Supplement 1. — eBox 1. Condition-Specific Enrollment Criteria eBox 2. Condition-Specific Treatment Plans eTable 1. Safer@Home Nurse Call Script eTable 2. Diagnostic/Procedural Criteria for Identification of Control Cohorts of Hospitalized Patients eTable 3. Definition of Cases of Cellulitis, Diabetic Foot Infection, and Pyelonephritis Presenting to Emergency Department (ED), by ICD-10-CM Codes eTable 4. Characteristics of Patients With “Core” Diagnoses eTable 5. Characteristics of Patients With Other Diagnoses eTable 6. Characteristics of Patients With Cellulitis eTable 7. Characteristics of Patients With Diabetic Foot Infections eTable 8. Characteristics of Patients With Osteomyelitis eTable 9. Characteristics of Patients With Pyelonephritis eTable 10. Characteristics of Patients With Bacterial Pneumonia eTable 11. Characteristics of Patients With COPD Exacerbation eTable 12. Characteristics of Patients With CHF eTable 13. Characteristics of Patients With Asthma eTable 14. Characteristics of Patients With COVID Pneumonia eTable 15. Characteristics of Patients With Acute Hypoxic Respiratory Failure Due to Viral infection eTable 16. Multivariable Linear Regression Model of Predictors of Observed Hospital Length of Stay (Days) eTable 17. Sensitivity Analysis, Observed Lengths of Stay for Enrollment and Matched Non-Enrollment Cohorts eTable 18. Hospital Admission Rates From Emergency Department for the Three Most Frequently Enrolled Safer@Home Diagnoses [file jamanetwopen-e2447352-s001.pdf]

Supplementary Online Content

Banerjee J, Lynch C, Gordon H, et al. Virtual home care for patients with acute illness. *JAMA Netw Open.* 2024;7(11):e2447352. doi:10.1001/jamanetworkopen.2024.47352

eBox 1. Condition-Specific Enrollment Criteria
eBox 2. Condition-Specific Treatment Plans
eTable 1. Safer@Home Nurse Call Script
eTable 2. Diagnostic/Procedural Criteria for Identification of Control Cohorts of Hospitalized Patients
eTable 3. Definition of Cases of Cellulitis, Diabetic Foot Infection, and Pyelonephritis Presenting to Emergency Department (ED), by ICD-10-CM Codes
eTable 4. Characteristics of Patients With "Core" Diagnoses
eTable 5. Characteristics of Patients With Other Diagnoses
eTable 6. Characteristics of Patients With Cellulitis
eTable 7. Characteristics of Patients With Diabetic Foot Infections
eTable 8. Characteristics of Patients With Osteomyelitis
eTable 9. Characteristics of Patients With Pyelonephritis
eTable 10. Characteristics of Patients With Bacterial Pneumonia
eTable 11. Characteristics of Patients With COPD Exacerbation
eTable 12. Characteristics of Patients With CHF
eTable 13. Characteristics of Patients With Asthma
eTable 14. Characteristics of Patients With COVID Pneumonia
eTable 15. Characteristics of Patients With Acute Hypoxic Respiratory Failure Due to Viral infection
eTable 16. Multivariable Linear Regression Model of Predictors of Observed Hospital Length of Stay (Days)
eTable 17. Sensitivity Analysis, Observed Lengths of Stay for Enrollment and Matched Non-Enrollment Cohorts
eTable 18. Hospital Admission Rates From Emergency Department for the Three Most Frequently Enrolled Safer@Home Diagnoses

This supplementary material has been provided by the authors to give readers additional information about their work.

© 2024 Banerjee J et al. *JAMA Network Open.*

eBox 1. Condition-Specific Enrollment Criteria

**Cellulitis Additional Criteria**<br>• No concern for necrotizing soft tissue infection, joint infection, and/or abscess(es) requiring drainage at the time of discharge<br>• No relevant history of multi-drug resistant organisms without adequate oral anti-microbial options. Consider contacting ID for assistance in complex cases.
**Diabetic Foot Infection (with or without Osteomyelitis) Additional Criteria**<br>• No concern for developing NSTI, joint infection, abscess(es) requiring drainage at the time of discharge, and/or acute limb ischemia<br>• No relevant history of multi-drug resistant organisms without adequate oral anti-microbial options. Consider contacting ID for assistance in complex cases. (Contact ID Stewardship Pharmacist during business hours and ID Consult Fellow during after-hours)<br>• Patient does not need MRI for definitive diagnosis prior to leaving acute care setting. Antbiotics can be started empirically, and MRI can be obtained in the outpatient setting. Safer@Home Team will connect patient to appropriate clinic/setting for definitive evaluation and management including MRI.
**Non-Spinal Osteomyelitis Additional Criteria**<br>• No concern for developing NSTI, joint infection, and/or abscess(es) requiring drainage at the time of discharge.<br>• No relevant history of multi-drug resistant organisms without adequate oral anti-microbial options.Consider contacting ID for assistance in complex cases. (Contact ID Stewardship Pharmacist during business hours and ID Consult Fellow during after-hours.<br>• Patient does not need MRI for definitive diagnosis prior to leaving acute care setting. Antibiotics can be started empirically, and MRI can be obtained in the outpatient setting. Safer@Home Team will connect Patient to appropriate clinic/setting for definitive evaluation and management including MRI.
**Spinal Osteomyelitis Additional Criteria**<br>• Negative blood cultures<br>• Appropriate imaging obtained (e.g. spine CT or MRI) to rule out the need for abscess drainage or surgical debridement<br>• No relevant history of multi-drug resistant organisms without adequate oral anti-microbial options. Consider contacting ID for assistance in complex cases. (Contact ID Stewardship Pharmacist during business hours and ID Consult Fellow during after-hours)<br>• Low/no suspicion for TB and fungal infection of the spine, or evaluated for these etiologies prior to discharge
**Pyelonephritis or Complicated UTI Additional Criteria**<br>• Inpatient urologic intervention not required at the time of discharge (e.g. obstructive uropathy, PCNT exchange, fistula)<br>• Not currently pregnant.<br>• No relevant history of multi-drug resistant organisms without adequate oral anti-microbial options. Consider contacting ID for assistance in complex cases. (Contact ID Stewardship Pharmacist during business hours and ID Consult Fellow during after-hours)<br>• No concern for renal abscess(es) that would require drainage (e.g. recurrent infection that relapsed after prior therapy)

• Patient has received 1 dose of either IV gentamicin or amikacin (if the patient has renal dysfunction, please contact pharmacy for dose adjustments) after first checking prior sensitivities before selecting therapy to cover for ESBL pathogens prior to discharge. If no IV access, can consider IM dosing in discussion with pharmacy.
**Bacterial Pneumonia Additional Criteria**<br>• Comfortable at rest and with minimal exertion (e.g. able to get out of bed, ambulate to bathroom and back)<br>• Stable oxygen saturation ≥92% with ≤3L/min of supplemental oxygen<br>• No relevant history of multi-drug resistant organisms without adequate oral anti-microbial options. Consider contacting ID for assistance in complex cases. (Contact ID Stewardship Pharmacist during business hours and ID Consult Fellow during after-hours)<br>• No indication for thoracentesis (e.g small or trace effusion not amenable for thoracentesis).<br>• Patient has received at least 1 dose of IV antibiotics targeted for treatment of pneumonia (i.e. ceftriaxone)
**COPD and Asthma Exacerbation Additional Criteria**<br>• Comfortable at rest and with minimal exertion (e.g., able to get out of bed, ambulate to bathroom and back)<br>• Stable oxygen saturation ≥92% with ≤3L/min of supplemental oxygen<br>• No evidence of worsening hypercapnia as determined by at least 2 serial VBGs
**Heart Failure Exacerbation Additional Criteria**<br>Inclusion Criteria:<br>• Established and verified history of chronic heart failure (i.e. prior TTE results)<br>• Symptoms secondary to mild heart failure exacerbation. Other causes have been ruled out.<br>• Comfortable at rest and with minimal exertion (e.g able to get out of bed, ambulate to bathroom and back)<br>• Stable oxygen saturation ≥90% on room air, or no increased oxygen requirement if on home O2 at baseline, after initial diuretic therapy.<br>• If the patient meets the above criteria but the provider still is not certain about whether the patient can be safely managed through Safer@Home, the following risk calculator can be used to help guide decision-making. Ottawa Heart Failure Risk Scale (OHFRS)<br><br>Exclusion Criteria:<br>• Worsening heart failure symptoms despite adherence to medications and in the absence of dietary indiscretion (i.e. consuming high sodium diet)<br>• Patient requiring > diuretic oral dose equivalent of Torsemide 80mg PO or Bumetanide 4mg PO per dose<br>• Concern for ACS (i.e. ischemic chest pain and/or positive troponin secondary to ACS)<br>• Presence of other causes of SOB/DOE (i.e. including but not limited to: advanced renal or liver disease, pulmonary hypertension, COPD, Asthma, or other chronic pulmonary disease)<br>• Active malignancy<br>• Anasarca<br>• Active substance use, impacting patient's ability to reliably follow up<br>• Dementia or altered mental status<br>• Other reasons for continued evaluation and management in the acute care setting (e.g Afib with RVR, AKI, acute valvular disease, thrombosis, encephalopathy, etc)<br>Adequate response to initial diuretic therapy:<br>• IV dose of loop diuretic (≥ 2x home po dose in IV form).<br>• UOP ≥1 – 1.5L within 2 hours of diuretic therapy

© 2024 Banerjee J et al. *JAMA Network Open.*

eBox 2. Condition-Specific Treatment Plans

Cellulitis Specific Treatment Plan

General

- If susceptibilities available, used targeted therapy (in consultation with ID if necessary)
- Duration of therapy is 5-6 days total
- If renal dysfunction present, adjust dose and discuss with pharmacy as needed.
- Even if patients have failed oral cephalexin or dicloxacillin, the below options may still be acceptable as failure may be caused by lack of adherence, failure to elevate the leg, low blood levels with short half-life of cephalexin and dicloxacillin, etc.

Without Abscess—principally target Strep.

- Cefadroxil 1 gm BID—preferred choice for cellulitis without an abscess. Cefadroxil easier to dose than tid/qid cephalexin.
- Linezolid 600 mg BID—consider if failed prior therapy or not a candidate for other options listed (requires calling ID for approval)
- TMP-SMX 1 DS BID—preferred for purulent cellulitis but is effective for non-purulent; be mindful of need for renal dose adjustment. If renal dysfunction, consider alternative medication or discuss with pharmacy regarding renal dosing.

With Abscess—principally target S. aureus

- TMP-SMX 1 DS BID—preferred for purulent cellulitis but is effective for non-purulent; be mindful of need for renal dose adjustment. If renal dysfunction, consider alternative medication or discuss with pharmacy regarding renal dosing.
- Linezolid 600 mg BID—consider if failed prior therapy or not a candidate for other options listed; requires calling ID for approval
- Cefadroxil 1 gm BID—only use if the organism is known to be MSSA or strep based on known culture data, as it has no coverage for MRSA

Diabetic Foot Infection Specific Treatment Plan

General

- If susceptibilities available, used targeted therapy (in consultation with ID if necessary)
- Duration of therapy:
- 5-6 days if exam is consistent with cellulitis4 weeks if concern for osteomyelitis but further workup needed (i.e. MRI as outpatient; antibiotic treatment can be extended in outpatient setting if needed)
- 4 weeks if concern for osteomyelitis but further workup needed (i.e. MRI as outpatient; antibiotic treatment can be extended in outpatient setting if needed)
- 6 weeks if findings consistent with osteomyelitis (i.e. XR findings consistent or able to probe to bone)
- If renal dysfunction present, adjust dose and discuss with pharmacy as needed.

If no abscess is present:

- Amoxicillin-clavulanic acid 875mg BID. If renal dysfunction, discuss with pharmacy regarding renal dosing.

If abscess is present (even if drained), or patient has previously grown MRSA:

- TMP-SMX 2 DS BID + metronidazole 1000mg daily (when using TMP-SMX, be mindful of need for renal dose adjustment. If renal dysfunction, consider alternative medication or discuss with pharmacy regarding renal dosing)
- Clindamycin 600mg TID —be aware of GI side effects and compliance

© 2024 Banerjee J et al. *JAMA Network Open.*

- Doxycycline 100 mg BID + metronidazole 500 mg bid

If patient has previously grown Pseudomonas (not resistant to ciprofloxacin/levofloxacin) or the wound is gangrenous:

- Levofloxacin 750 mg daily + metronidazole 1000mg daily. If renal dysfunction, discuss with pharmacy regarding renal dosing for levofloxacin.

Non-Spinal Osteomyelitis Specific Treatment Plan

General:

- If susceptibilities available, used targeted therapy (in consultation with ID if necessary)
- Duration of therapy: 6 weeks total
- If renal dysfunction present, adjust dose and discuss with pharmacy as needed.
- When considering the options below, only use rifampin in combination and never as monotherapy.

Antibiotics:

- TMP-SMX 2 DS BID—(when using TMP-SMX, be mindful of need for renal dose adjustment. If renal dysfunction, consider alternative medication or discuss with pharmacy regarding renal dosing)
- Clindamycin 600 mg TID—be aware of GI side effects and compliance
- Levofloxacin 750 mg daily +/- rifampin 600 mg daily—add rifampin if Gram positive likely, not needed if Gram negative likely, ensure no drug interactions if considering rifampin. If renal dysfunction, discuss with pharmacy regarding renal dosing for levofloxacin.
- Doxycycline 100 mg BID rifampin 600 mg daily—less data for doxycycline than other options, ensure no drug interactions if considering rifampin

Spinal Osteomyelitis Specific Treatment Plan

General

- If susceptibilities available, use target therapy (in consultation with ID if necessary)
- Duration of therapy: 6 weeks total
- If renal dysfunction present, adjust dose and discuss with pharmacy as needed.
- When considering the options below, only use rifampin in combination and never as monotherapy.

Antibiotic options:

- TMP-SMX 2 DS BID—(when using TMP-SMX, be mindful of need for renal dose adjustment. If renal dysfunction, consider alternative medication or discuss with pharmacy regarding renal dosing)
- Clindamycin 600mg TID —be aware of GI side effects and compliance
- Levofloxacin 750 mg daily +/- rifampin 600 mg daily—add rifampin if Gram positive likely, not needed if Gram negative likely, ensure no drug interactions if considering rifampin. If renal dysfunction, discuss with pharmacy regarding renal dosing for levofloxacin.
- Doxycycline 100 mg BID +/- rifampin 600 mg daily—less data for doxycycline than other options, ensure no drug interactions if considering rifampin

Pyelonephritis or Complicated UTI Specific Treatment Plan

General

- If susceptibilities available, used targeted therapy (in consultation with ID if necessary)
- Duration of therapy is 5-7 days
- Administer 1 dose IV gentamicin or amikacin before discharging patient—if the patient has had recurrent UTIs or has grown an ESBL organism in the past, make sure to check prior sensitivities before selecting therapy. Plan to administer gentamicin unless patient has prior history of gentamicin resistance. If prior gentamicin resistance, can give amikacin.

© 2024 Banerjee J et al. *JAMA Network Open.*

- If renal dysfunction present, adjust dose and discuss with pharmacy as needed.

Antibiotic options:

- Levofloxacin 750 mg daily (requires less frequent dosing than ciprofloxacin). If renal dysfunction, discuss with pharmacy regarding renal dosing for levofloxacin.
- TMP-SMX 1 DS BID. If renal dysfunction, consider alternative medication or discuss with pharmacy regarding renal dosing.
- Fosfomycin 3 gm daily—only use if specific concern for ESBL or MDR pathogen resistant to levofloxacin or TMP-SMX

Bacterial Pneumonia Specific Treatment Plan

General

- If susceptibilities available, used targeted therapy (in consultation with ID if necessary)
- Duration of therapy: 3-5 days total
- If renal dysfunction present, adjust dose and discuss with pharmacy as needed.

Antibiotics

- Amoxicillin 1000 mg TID If renal dysfunction, discuss with pharmacy regarding renal dosing.
- Azithromycin 500mg qday
- Doxycycline 100 mg BID
- Levofloxacin 750 mg daily—only use if there is a concern for Gram negative pathogens (e.g., healthcare exposure). If renal dysfunction, discuss with pharmacy regarding renal dosing.

Heart Failure Exacerbation Specific Treatment Plan

General guidance for Prescription Diuretic Dosing at Time of Discharge/Safer@Home Referral

- Choice of Diuretic:
    - If on Furosemide at home, discontinue furosemide and switch to Torsemide
    - If on Torsemide at home, continue to use Torsemide
    - If on Bumetanide at home, continue to use Bumetanide

- If mild edema on exam:
    - Double the current home oral dose of diuretic for 3 days after discharge, then go back to prior home oral dose.
- If moderate edema on exam:
    - Double the current home oral dose of diuretic for 7 days after discharge, then go back to prior home oral dose.
- Please prescribe a 1 month supply of diuretic medication
- Of note, the Safer@Home Team can adjust diuretic dosing as needed after discharge.

Diuretic Conversion Ratios

Furosemide PO → Torsemide PO	2:1	e.g. Furosemide 40mg PO = Torsemide 20mg PO
Furosemide IV → Torsemide PO	1:1	e.g. Furosemide 40mg IV = Torsemide 40mg PO
Bumetanide IV → Bumetanide PO	1:1	e.g. Bumetanide 1mg IV= Bumetanide 1mg PO

Examples of Discharging Diuretic Regimens per Recommendations Above:

(Patient's Home Dose)	(Edema)	(Discharging Diuretic Regimen)
Furosemide 20mg PO BID	Mild	Torsemide 20mg PO BID for 3 days, then Torsemide 20mg PO qday
Furosemide 20mg PO BID	Mod.	Torsemide 20mg PO BID for 7 days, then Torsemide 20mg PO qday
Furosemide 20mg PO qday	Mild	Torsemide 20mg PO qday for 3 days, then Torsemide 10mg PO qday

© 2024 Banerjee J et al. *JAMA Network Open.*

Furosemide 20mg PO qday	Mod.	Torsemide 20mg PO qday for 7 days, then Torsemide 10mg PO qday
Furosemide 40mg PO BID	Mild	Torsemide 40mg PO BID for 3 days, then Torsemide 40mg PO qday
Furosemide 40mg PO BID	Mod.	Torsemide 40mg PO BID for 7 days, then Torsemide 40mg PO qday
Bumetanide 1mg PO BID	Mild	Bumetanide 2mg PO BID for 3 days, then Bumetanide 1mg PO BID
Bumetanide 1mg PO BID	Mod.	Bumetanide 2mg PO BID for 7 days, then Bumetanide 1mg PO BID
Bumetanide 2mg PO BID	Mild	Bumetanide 4mg PO BID for 3 days, then Bumetanide 2mg PO BID
Bumetanide 2mg PO BID	Mod.	Bumetanide 4mg PO BID for 7 days, then Bumetanide 2mg PO BID

© 2024 Banerjee J et al. *JAMA Network Open.*

eTable 1. Safer@Home Nurse Call Script

**General Questions** (for all Safer @ Home patients)	**Question**	**Response (Y/N)**	**Triage/Referral Guidance (if yes)**
	Febrile (temp> 38°C) since last call/visit?		UC
	Heart rate>110 (persistently over 15 minutes currently or last few readings)		ED
	New or worsening dizziness/lightheadedness?		UC
	Episode of syncope/LOC?		ED
	Severe pain not controlled with prn home medications?		UC
	Vomiting or worsening nausea causing patient unable to tolerate oral medications?		ED
	Generally feeling more unwell, not improving, and would like to be seen by a provider?		UC
	Compliant with medications (medications obtain and taking as prescribed)?		If **no**, please provide counseling and education
	Issues or questions with home DME?		If **yes**, please provide education and support/triage as needed.

**Disease Specific Questions** (ask as appropriate)	**Disease**	**Question(s)**	**Response (Y/N)**	**Triage/Referral Guidance (if yes)**
	Cellulitis	Worsening pain at the site of infection?		UC
		Worsening redness/swelling at site of infection?		• If worsening, refer to UC.<br>• If stable but not improving, repeat phone call next day (if not improving over course of 3 days, refer to UC)
		If limb involvement, is patient elevating the appropriate limb?		If no, provide education and counseling
	Diabetic Foot Infection/	Worsening pain at the site of infection?		UC

© 2024 Banerjee J et al. *JAMA Network Open.*

	Diabetic Osteo	New or worsening redness and swelling at site of infection?		UC
		When patient palpates/squeezes the infected area, does pus/yellow fluid come out, and if yes, is the amount increased from the day prior? (please ask patient to do this during the phone interview if not recently assessed that day)		UC
	Spinal Osteomyelitis	New or increased:<br>- Numbness in perineal region<br>- Lower extremity weakness		ED
		New urinary or bowel incontinence		ED
		Worsening pain at the site of infection?		UC
	Pyelonephritis	Significantly worsening flank pain?		UC
	Pneumonia (Bacterial, COVID-19, Viral)	Worsening shortness of breath?		UC
		New or significantly worsening chest pain/discomfort (not associated with cough)?		UC
		Increased oxygen requirements with saturation >90% on 5L NC?		ED

© 2024 Banerjee J et al. *JAMA Network Open.*

		Increased oxygen requirements with saturation <90% on 5L NC?		ED (advise patient to call 911)
	COPD and Asthma	Worsening shortness of breath?		UC
		New or significantly worsening chest pain (not associated with cough)?		UC
		Increased oxygen requirements with saturation >90% on 5L NC?		ED
		Increased oxygen requirements with saturation <90% on 5L NC?		ED (advise patient to call 911)
	Heart Failure Exacerbation	Worsening shortness of breath or uncomfortable breathing at rest/ with minimal exertion?		ED
		New or worsening chest pain or discomfort (not associated with cough)?		ED or UCC (discuss with attending)
		Increased discomfort or increased swelling in the lower body?		UCC
		Discomfort or increased swelling in the abdomen?		UCC
		Increased fatigue?		UCC

© 2024 Banerjee J et al. *JAMA Network Open.*

		Increased trouble sleeping due to shortness of breath or need to prop self up more to reduce shortness of breath? *(Using more pillows or prop self up more at night?)*		UCC
		New sensation of palpitations or rapid heartbeat?		UCC
		Increased oxygen requirement (either from baseline or requirement at time or discharge/referral)		ED (Call 911 if SpO2<90% if on maximum oxygen settings)
		Weight gain (>3 pounds in 24 hour period or > 5 pounds over 1 week)		UCC

© 2024 Banerjee J et al. *JAMA Network Open.*

eTable 2. Diagnostic/Procedural Criteria for Identification of Control Cohorts of Hospitalized Patients

Diagnosis	Coding Criteria
Cellulitis	A AND (B OR C) AND D AND E where:<br>A = Any Dx ICD-10 (b950, H0501, H05011, H05012, H05013, H05019, H601, H6010, H6011, H6012, H6013, h44001, K122, L03, L030, L0301, L03011, L03012, L03019, L0303, L03031, L03032, L03039, L031, L0311, L03111, L03112, L03113, L03114, L03115, L03116, L03119, L032, L0321, L03211, L03213, L0322, L03221, L033, L0331, L03311, L03312, L03313, L03314, L03315, L03316, L03317, L03319, L03321, L038, L0381, L03811, L03818, L039, L0390, N4822, N730, N731, N732, L983, l03211, l0231, l02215, n492)<br>B = MS-DRG (121 Acute major eye infections w cc/mcc, 122 Acute major eye infections w/o cc/mcc, 602 Cellulitis w mcc, 603 Cellulitis w/o mcc, 727 Inflammation of the male reproductive system w mcc, 728 Inflammation of the male reproductive system w/o mcc, 871 Septicemia or severe sepsis w/o mv >96 hours w mcc, 872 Septicemia or severe sepsis w/o mv >96 hours w/o mcc)<br>C = Principal Diagnosis ICD-10(r221, t8579xa, a4101, a4102, j0190, l03115, m272)<br>D = Any Diagnosis Not ICD-10(E0852, E1052, E1152, E10621, E11621, e11628, e1169, i38, i469, U071, j9601, j9622, r532, m868x6, m869, i214, i21a1, j189, j2699, k831, r6521, s22068a, g8221, q225, k7682, k767, z651, k8520, g039, t402x1a, m726, s0219xb, s02641a, g936, c8113, w57xxxa, 8441, k920, h04001, h04301, s90862a, n4821, s70361a, b309, t83511a, n454, m60009, a4289, s82201a, w540xxa, m60822, l738)<br>E = Any Procedure Not ICD-10 (0bh17ez, 0bh18ez, 3E033XZ, 5a1d70z, 5a09457, 09tu8zz, 0dbp8zx, 0vb5xzz)
Diabetic Foot Infection	((A AND B) OR ((C AND D) OR (E AND F AND G))) AND H AND I, where:<br>A = Principal Diagnosis ICD-10(e11621, e10621)<br>B = Secondary Diagnosis ICD-10(l97529, l02611, l02612, l03115, l03116, l97516, l97519, l97523, m86172, m868x7, m869, l97419)<br>C = Principal Diagnosis ICD-10(e1152, e11628, e1169, l03116, l02611, l02612, m869, t8744)<br>D = Secondary Diagnosis ICD-10(e10621, e1152, e11621, e1169, l02611, l98498, m86172, m86171)<br>E = Principal Diagnosis ICD-10(a419, a400, a401, a409, a4101, a4102)<br>F = Secondary Diagnosis ICD-10(e10621, e11621, e1152, e1169)<br>G = Secondary Diagnosis ICD-10(l02611, l03115, l03116, l02612, m86172, m86671, m86672, l97519, l97529)<br>H = Any Diagnosis Not ICD-10(i330, g062, z651, m009, z9089, h3340, f1491, r0902, i82532, j189, s82192a, j129, i69313, i5040, a599, i5023, t782xxa, r6521, m9271, r319, i429, f0390, i452, m5116, f0390, 041l09n, i452, g309)

© 2024 Banerjee J et al. *JAMA Network Open.*

	I = Any Procedure Not ICD-10(0y6c0z1, 0y6h0z1, 0y6h0z2, 0y6m0z9, 0y6m0zb, 0y6m0zc, 0y6m0zd, 0y6m0zf, 0y6p0z0, 0y6m0z, 0y6n0z9, 0y6n0zb, 0y6n0zc, 0y6n0zd, 0y6n0zf, 0y6h0z3, 0y6j0z3, 027136z, 4a023n6, b41gyzz, 047q3zz, 047n3zz, 3e033xz, 0qs906z, 0y6j0z1, 0qbn0zz, 0bh17ez, 0bh18ez, 3E033XZ, 0qbq0zz, 0qdn0zz, 027034z, 3e0c3gc, 041k09l)
Osteomyelitis	A AND (B OR (C AND D)), where:<br>A = Any Diagnosis Not ICD-10(E0852, E1052, E1152, E10621, E11621, k8309, s62522b, i21a1, i330, l89154, l89159, i38, s62521b, i69351, j850, s82091b, i059, g062, l89324, a1801, a1889, m20002, l89153, s62607b, a752, s42461b, c3411, b967, j9602, z66, e1151, s82291b, l97519, t25222d, m87851, c20, z944, i2699, s52371b, e1169, l89314, s92241b, s62392b, s42022b, l100, u071, k56609, s82891b, s02651b, i459, s0266xb, f1113, m84641a, s82872b, z651, f29, b457, f209, f1513, s51812a, c9591, s0219xb, s02652b, f1911, y838, s0266xa, s62512b, s52692b, m87852, f1510, s6832b, s32302b, s82891a, s020xxb, g060, s82292b, f1410, s02119b, s06a1xa, l03012, m86642, i81, c8585, s82001b, s82002b, s82202c, s2242xb, s02641b, k651, s82871b, s066x0a, s52272b, s61011a, f13239, s42301b, k750, j181, s0231xb, s62211b, s72391b, g039, s42492b, s62600b, j45901, z98890, i82411, s32601b, s42401b, s62391, s82491b, s42031b, s42302b, s32392b, s62393b, s72392b, l89214, l89224, y831, t8131xa, s36031a, s42202b, s02411b, n390)<br>B = Principal Diagnosis ICD-10(A0105, A0224, A5443, H0502, H05021, H05022, H05023, H05029, M462, M4620, M4621, M4622, M4623, M4624, M4625, M4626, M4627, M4628, M86, M860, M8600, M8601, M86011, M86012, M86019, M8602, M86021, M86022, M86029, M8603, M86031, M86032, M86039, M8604, M86041, M86042, M86049, M8605, M86051, M86052, M86059, M8606, M86061, M86062, M86069, M8607, M86071, M86072, M86079, M8608, M8609, M861, M8610, M8611, M86111, M86112, M86119, M8612, M86121, M86122, M86129, M8613, M86131, M86132, M86139, M8614, M86141, M86142, M86149, M8615, M86151, M86152, M86159, M8616, M86161, M86162, M86169, M8617, M86171, M86172, M86179, M8618, M8619, M862, M8620, M8621, M86211, M86212, M86219, M8622, M86221, M86222, M86229, M8623, M86231, M86232, M86239, M8624, M86241, M86242, M86249, M8625, M86251, M86252, M86259, M8626, M86261, M86262, M86269, M8627, M86271, M86272, M86279, M8628, M8629, M863, M8630, M8631, M86311, M86312, M86319, M8632, M86321, M86322, M86329, M8633, M86331, M86332, M86339, M8634, M86341, M86342, M86349, M8635, M86351, M86352, M86359, M8636, M86361, M86362, M86369, M8637, M86371, M86372, M86379, M8638, M8639, M864, M8640, M8641, M86411, M86412, M86419, M8642, M86421, M86422, M86429, M8643, M86431, M86432, M86439, M8644, M86441, M86442, M86449, M8645, M86451, M86452, M86459, M8646, M86461, M86462, M86469,

© 2024 Banerjee J et al. *JAMA Network Open.*

	M8647, M86471, M86472, M86479, M8648, M8649, M865, M8650, M8651, M86511, M86512, M86519, M8652, M86521, M86522, M86529, M8653, M86531, M86532, M86539, M8654, M86541, M86542, M86549, M8655, M86551, M86552, M86559, M8656, M86561, M86562, M86569, M8657, M86571, M86572, M86579, M8658, M8659, M866, M8660, M8661, M86611, M86612, M86619, M8662, M86621, M86622, M86629, M8663, M86631, M86632, M86639, M8664, M86641, M86642, M86649, M8665, M86651, M86652, M86659, M8666, M86661, M86662, M86669, M8667, M86671, M86672, M86679, M8668, M8669, M868, M868X, M868X0, M868X1, M868X2, M868X3, M868X4, M868X5, M868X6, M868X7, M868X8, M868X9, M869, S020XXB, S02101B, S02102B, S02109B, S02110B, S02111B, S02112B, S02113B, S02118B, S02119B, S0211AB, S0211BB, S0211CB, S0211DB, S0211EB, S0211FB, S0211GB, S0211HB, S02121B, S02122B, S02129B, S0219XB, S022XXB, S0230XB, S0231XB, S0232XB, S023XXB, S02400B, S02401B, S02402B, S0240AB, S0240BB, S0240CB, S0240DB, S0240EB, S0240FB, S02411B, S02412B, S02413B, S0242XB, S025XXB, S02602B, S02609B, S02611B, S02612B, S02621B, S02622B, S02631B, S02632B, S02640B, S02641B, S02642B, S0264XB, S02650B, S02651B, S02652B, S0265XB, S0266XB, S02670B, S02671B, S02672B, S0269XB, S02831B, S02832B, S02839B, S02841B, S02842B, S02849B, S0285XB, S0291XB, S0292XB, S1201XB, S1202XB, S12090B, S12110B, S12111B, S12112B, S12120B, S12121B, S12190B)S12290B, S12390B, S12490B, S12590B, S12690B, S22008B, S22009B, S22010B, S22011B, S22012B, S22018B, S22019B, S22021B, S22022B, S22028B, S22029B, S22030B, S22031B, S22032B, S22038B, S22039B, S22041B, S22042B, S22048B, S22049B, S22050B, S22051B, S22052B, S22058B, S22059B, S22060B, S22061B, S22062B, S22068B, S22069B, S22070B, S22071B, S22072B, S22078B, S22079B, S22080B, S22081B, S22082B, S22088B, S22089B, S2220XB, S2221XB, S2222XB, S2223XB, S2224XB, S2231XB, S2232XB, S2239XB, S2241XB, S2242XB, S2243XB, S2249XB, S225XXB, S229XXB, S32001B, S32008B, S32009B, S32010B, S32011B, S32012B, S32018B, S32019B, S32020B, S32021B, S32022B, S32028B, S32029B, S32030B, S32031B, S32032B, S32038B, S32039B, S32040B, S32041B, S32042B, S32048B, S32049B, S32050B, S32051B, S32052B, S32058B, S32059B, S3210XB, S32110B, S32111B, S32112B, S32119B, S32120B, S32121B, S32122B, S32129B, S32130B, S32131B, S32132B, S32139B, S3214XB, S3215XB, S3216XB, S3217XB, S3219XB, S322XXB, S32301B, S32302B, S32309B, S32311B, S32312B, S32313B, S32314B, S32315B, S32316B, S32391B, S32392B, S32399B, S32401B, S32402B, S32409B, S32451B, S32452B, S32454B, S32455B, S32471B, S32472B, S32481B, S32482B, S32483B, S32484B, S32485B, S32486B, S32491B, S32492B, S32499B, S32501B, S32502B, S32509B, S32511B, S32512B, S32519B,

© 2024 Banerjee J et al. *JAMA Network Open.*

	S32591B, S32592B, S32599B, S32601B, S32602B, S32609B, S32611B, S32612B, S32613B, S32614B, S32615B, S32616B, S32691B, S32692B, S32699B, S3289XB, S42001B, S42002B, S42009B, S42018B, S42021B, S42022B, S42023B, S42024B, S42025B, S42031B, S42032B, S42035B, S42111B, S42112B, S42122B, S42132B, S42151B, S42152B, S42191B, S42192B, S42201B, S42202B, S42231B, S42232B, S42241B, S42242B, S42292B, S42301B, S42302B, S42391B, S42392B, S42399B, S42401B, S42402B, S42452B, S42461B, S42462B, S42471B, S42472B, S42474B, S42475B, S42492B, S4291XB, S4292XB, S52092B, S52271B, S52272B, S52279B, S52281B, S52281C, S52281M, S52281Q, S52282B, S52282C, S52282M, S52282Q, S52283B, S52291B, S52292B, S52371B, S52372B, S52381B, S52381C, S52382B, S52382C, S52389B, S52531B, S52532B, S52539B, S52541B, S52542B, S52549B, S52561B, S52562B, S52569B, S52692B, S5291XB, S5292XB, S62101B, S62102B, S62121B, S62122B, S62123B, S62161B, S62162B, S62163B, S62164B, S62165B, S62166B, S62211B, S62212B, S62213B, S62221B, S62222B, S62223B, S62224B, S62225B, S62226B, S62291B, S62292B, S62308B, S62309B, S62318B, S62328B, S62338B, S62390B, S62391B, S62392B, S62393B, S62394B, S62395B, S62396B, S62397B, S62398B, S62399B, S62501B, S62502B, S62509B, S62511B, S62512B, S62521B, S62522B, S62525B, S62600B, S62601B, S62603B, S62604B, S62605B, S62607B, S62608B, S62609B, S62628B, S62638B, S6290XB, S6291XB, S6292XB, S72391B, S72392B, S728X1B, S728X2B, S728X2C, S728X9B, S7291XB, S7292XB, S82001B, S82002B, S82091B, S82092B, S82099B, S82111B, S82112B, S82291B, S82292B, S82491B, S82492B, S82871B, S82872B, S82891B, S82892B, S82899B, S8291XB, S8292XB, S92001B, S92002B, S92009B, S92011B, S92012B, S92013B, S92014B, S92015B, S92061B, S92062B, S92101B, S92102B, S92109B, S92111B, S92112B, S92113B, S92114B, S92115B, S92116B, S92121B, S92122B, S92123B, S92124B, S92125B, S92126B, S92132B, S92141B, S92142B, S92143B, S92144B, S92145B, S92146B, S92191B, S92192B, S92199B, S92201B, S92202B, S92211B, S92212B, S92213B, S92214B, S92215B, S92221B, S92222B, S92241B, S92242B, S92302B, S92401B, S92402B, S92404B, S92405B, S92491B, S92492B, S92499B, S92501B, S92502B, S92591B, S92592B, S92599B, S92811B, S92812B, S92819B, S92901B, S92902B, S92909B, S92911B, S92912B, S92919B, S99001B, S99002B, S99009B, S99091B, S99092B, S99099B, S99101B, S99102B, S99191B, S99192B, S99199B, S99201B, S99202B, S99291B, S99292B, S99299B)<br>C = MS-DRG (856 Postoperative or post-traumatic infections w o.r. proc w mcc, 857 Postoperative or post-traumatic infections w o.r. proc w cc, 858 Postoperative or post-traumatic infections w o.r. proc w/o cc/mcc, 871 Septicemia or severe sepsis w/o mv >96

© 2024 Banerjee J et al. *JAMA Network Open.*

	hours w mcc, 872 Septicemia or severe sepsis w/o mv >96 hours w/o mcc)<br>D = Any Diagnosis ICD-10(A0105, A0224, A5443, H0502, H05021, H05022, H05023, H05029, M462, M4620, M4621, M4622, M4623, M4624, M4625, M4626, M4627, M4628, M86, M860, M8600, M8601, M86011, M86012, M86019, M8602, M86021, M86022, M86029, M8603, M86031, M86032, M86039, M8604, M86041, M86042, M86049, M8605, M86051, M86052, M86059, M8606, M86061, M86062, M86069, M8607, M86071, M86072, M86079, M8608, M8609, M861, M8610, M8611, M86111, M86112, M86119, M8612, M86121, M86122, M86129, M8613, M86131, M86132, M86139, M8614, M86141, M86142, M86149, M8615, M86151, M86152, M86159, M8616, M86161, M86162, M86169, M8617, M86171, M86172, M86179, M8618, M8619, M862, M8620, M8621, M86211, M86212, M86219, M8622, M86221, M86222, M86229, M8623, M86231, M86232, M86239, M8624, M86241, M86242, M86249, M8625, M86251, M86252, M86259, M8626, M86261, M86262, M86269, M8627, M86271, M86272, M86279, M8628, M8629, M863, M8630, M8631, M86311, M86312, M86319, M8632, M86321, M86322, M86329, M8633, M86331, M86332, M86339, M8634, M86341, M86342, M86349, M8635, M86351, M86352, M86359, M8636, M86361, M86362, M86369, M8637, M86371, M86372, M86379, M8638, M8639, M864, M8640, M8641, M86411, M86412, M86419, M8642, M86421, M86422, M86429, M8643, M86431, M86432, M86439, M8644, M86441, M86442, M86449, M8645, M86451, M86452, M86459, M8646, M86461, M86462, M86469, M8647, M86471, M86472, M86479, M8648, M8649, M865, M8650, M8651, M86511, M86512, M86519, M8652, M86521, M86522, M86529, M8653, M86531, M86532, M86539, M8654, M86541, M86542, M86549, M8655, M86551, M86552, M86559, M8656, M86561, M86562, M86569, M8657, M86571, M86572, M86579, M8658, M8659, M866, M8660, M8661, M86611, M86612, M86619, M8662, M86621, M86622, M86629, M8663, M86631, M86632, M86639, M8664, M86641, M86642, M86649, M8665, M86651, M86652, M86659, M8666, M86661, M86662, M86669, M8667, M86671, M86672, M86679, M8668, M8669, M868, M868X, M868X0, M868X1, M868X2, M868X3, M868X4, M868X5, M868X6, M868X7, M868X8, M868X9, M869, S020XXB, S02101B, S02102B, S02109B, S02110B, S02111B, S02112B, S02113B, S02118B, S02119B, S0211AB, S0211BB, S0211CB, S0211DB, S0211EB, S0211FB, S0211GB, S0211HB, S02121B, S02122B, S02129B, S0219XB, S022XXB, S0230XB, S0231XB, S0232XB, S023XXB, S02400B, S02401B, S02402B, S0240AB, S0240BB, S0240CB, S0240DB, S0240EB, S0240FB, S02411B, S02412B, S02413B, S0242XB, S025XXB, S02602B, S02609B, S02611B, S02612B, S02621B, S02622B, S02631B, S02632B, S02640B, S02641B, S02642B, S0264XB, S02650B, S02651B, S02652B, S0265XB, S0266XB, S02670B, S02671B,

© 2024 Banerjee J et al. *JAMA Network Open.*

	S02672B, S0269XB, S02831B, S02832B, S02839B, S02841B, S02842B, S02849B, S0285XB, S0291XB, S0292XB, S1201XB, S1202XB, S12090B, S12110B, S12111B, S12112B, S12120B, S12121B, S12190B)S12290B, S12390B, S12490B, S12590B, S12690B, S22008B, S22009B, S22010B, S22011B, S22012B, S22018B, S22019B, S22021B, S22022B, S22028B, S22029B, S22030B, S22031B, S22032B, S22038B, S22039B, S22041B, S22042B, S22048B, S22049B, S22050B, S22051B, S22052B, S22058B, S22059B, S22060B, S22061B, S22062B, S22068B, S22069B, S22070B, S22071B, S22072B, S22078B, S22079B, S22080B, S22081B, S22082B, S22088B, S22089B, S2220XB, S2221XB, S2222XB, S2223XB, S2224XB, S2231XB, S2232XB, S2239XB, S2241XB, S2242XB, S2243XB, S2249XB, S225XXB, S229XXB, S32001B, S32008B, S32009B, S32010B, S32011B, S32012B, S32018B, S32019B, S32020B, S32021B, S32022B, S32028B, S32029B, S32030B, S32031B, S32032B, S32038B, S32039B, S32040B, S32041B, S32042B, S32048B, S32049B, S32050B, S32051B, S32052B, S32058B, S32059B, S3210XB, S32110B, S32111B, S32112B, S32119B, S32120B, S32121B, S32122B, S32129B, S32130B, S32131B, S32132B, S32139B, S3214XB, S3215XB, S3216XB, S3217XB, S3219XB, S322XXB, S32301B, S32302B, S32309B, S32311B, S32312B, S32313B, S32314B, S32315B, S32316B, S32391B, S32392B, S32399B, S32401B, S32402B, S32409B, S32451B, S32452B, S32454B, S32455B, S32471B, S32472B, S32481B, S32482B, S32483B, S32484B, S32485B, S32486B, S32491B, S32492B, S32499B, S32501B, S32502B, S32509B, S32511B, S32512B, S32519B, S32591B, S32592B, S32599B, S32601B, S32602B, S32609B, S32611B, S32612B, S32613B, S32614B, S32615B, S32616B, S32691B, S32692B, S32699B, S3289XB, S42001B, S42002B, S42009B, S42018B, S42021B, S42022B, S42023B, S42024B, S42025B, S42031B, S42032B, S42035B, S42111B, S42112B, S42122B, S42132B, S42151B, S42152B, S42191B, S42192B, S42201B, S42202B, S42231B, S42232B, S42241B, S42242B, S42292B, S42301B, S42302B, S42391B, S42392B, S42399B, S42401B, S42402B, S42452B, S42461B, S42462B, S42471B, S42472B, S42474B, S42475B, S42492B, S4291XB, S4292XB, S52092B, S52271B, S52272B, S52279B, S52281B, S52281C, S52281M, S52281Q, S52282B, S52282C, S52282M, S52282Q, S52283B, S52291B, S52292B, S52371B, S52372B, S52381B, S52381C, S52382B, S52382C, S52389B, S52531B, S52532B, S52539B, S52541B, S52542B, S52549B, S52561B, S52562B, S52569B, S52692B, S5291XB, S5292XB, S62101B, S62102B, S62121B, S62122B, S62123B, S62161B, S62162B, S62163B, S62164B, S62165B, S62166B, S62211B, S62212B, S62213B, S62221B, S62222B, S62223B, S62224B, S62225B, S62226B, S62291B, S62292B, S62308B, S62309B, S62318B, S62328B, S62338B, S62390B, S62391B, S62392B, S62393B, S62394B, S62395B, S62396B, S62397B, S62398B, S62399B, S62501B,

© 2024 Banerjee J et al. *JAMA Network Open.*

	S62502B, S62509B, S62511B, S62512B, S62521B, S62522B, S62525B, S62600B, S62601B, S62603B, S62604B, S62605B, S62607B, S62608B, S62609B, S62628B, S62638B, S6290XB, S6291XB, S6292XB, S72391B, S72392B, S728X1B, S728X2B, S728X2C, S728X9B, S7291XB, S7292XB, S82001B, S82002B, S82091B, S82092B, S82099B, S82111B, S82112B, S82291B, S82292B, S82491B, S82492B, S82871B, S82872B, S82891B, S82892B, S82899B, S8291XB, S8292XB, S92001B, S92002B, S92009B, S92011B, S92012B, S92013B, S92014B, S92015B, S92061B, S92062B, S92101B, S92102B, S92109B, S92111B, S92112B, S92113B, S92114B, S92115B, S92116B, S92121B, S92122B, S92123B, S92124B, S92125B, S92126B, S92132B, S92141B, S92142B, S92143B, S92144B, S92145B, S92146B, S92191B, S92192B, S92199B, S92201B, S92202B, S92211B, S92212B, S92213B, S92214B, S92215B, S92221B, S92222B, S92241B, S92242B, S92302B, S92401B, S92402B, S92404B, S92405B, S92491B, S92492B, S92499B, S92501B, S92502B, S92591B, S92592B, S92599B, S92811B, S92812B, S92819B, S92901B, S92902B, S92909B, S92911B, S92912B, S92919B, S99001B, S99002B, S99009B, S99091B, S99092B, S99099B, S99101B, S99102B, S99191B, S99192B, S99199B, S99201B, S99202B, S99291B, S99292B, S99299B, t8143xa)
Pyelonephritis/cUTI	((A AND C) OR (A AND D) OR (A AND F) OR (A AND I) OR (B AND C) OR (B AND D) OR (D AND F) OR (C AND H)) AND E AND G, where:<br>A = Principal Diagnosis ICD-10(N10, N111, n12, n390, n3080, N2885, N136, N151, t83512a)<br>B = Principal Diagnosis ICD-10(a4151, a4159, r7881)<br>C = Secondary Diagnosis ICD-10(n12, n136, n16, n3000, n3001, n3040, n390, z960, b961, b9620, q059, t83092a, b9689, n200, n202, r7881, z87440, N10, N111, n3080, N2885, N136, N151, t83512a, Z936)<br>D = MS-DRG (689 Kidney & urinary tract infections w mcc, 690 Kidney & urinary tract infections w/o mcc, 695 Kidney & urinary tract signs & symptoms w mcc, 696 Kidney & urinary tract signs & symptoms w/o mcc, 698 Other kidney & urinary tract diagnoses w mcc, 699 Other kidney & urinary tract diagnoses w cc)<br>E = Any Diagnosis Not ICD-10(J690, k861, b451, r180, b387, j80, c7989, m4628, n823, i69351, t8141xa, f1999, l0211, z515, j9612, i428, C801, a159, s065x0a, d5700, h44011, s01111a, c561, r29707, z89442, i314, r262, k754, i82b22, m353, v892xxs, j159, i69354, j982, l02416, 426x6a, z85528, i5031, m3500, z95810, k8000, m4626, l03315, s2193xs, i213, g912, j302, m25552, i76, k592, z853, j129, m8618, e0590, z634, w138xxd, i319, c218, r161, t82598a, e042, d5702, c8591, y908, d6109, d3500, a759, k7011, k631, c50912, t463x5a, s0990xa, i82401, i69954, c787, t8092xa, k5939, i052, a539, s065xaa, q044, i454, t730xxa, f3130, k831, r471, h5702, h3552, m303, i501, i3139, s069x9a, i469, i4720, i2692, j1282, l97513, l853, m25561, m1a9xx0, i71010, m5430,

	n2886, e807, m8448xa, e272, i63522K767, g9520, m24532, d6481, f10159, i639, i81, t7401xa, n35919, r571, k469, d57419, j9602, l03115, e870, k56600, i442, e883, i952, r61, i69320, k9622, r402112, r4701)<br>F = Any Diagnosis ICD-10(a419, a4151, n134, n3091, n390, b961, b964)<br>G = Any Procedure Not ICD-10(009u3zx, 06183j4, 0bh17ez, 0bh18ez, 0d9w30z, 0w9h30z, 0dtj4zz, 0f9030z, 0ft40zz, 0ft44zz, 0j980zz, 0t733dz, 0t738dz, 0t768dz, 0t778dz, 0t9430z, 0tb10zx, 0w110jg, 0w9g00z, 0w9g3zz, 0wjg0zz, 0wpg3yz, 3e1m39z, 4a023n7, 5a1d90z, bt131zz, 0f798dz, 5a09357, 0djd8zz, 0hq1xzz)<br>H = MS-DRG (870 Septicemia or severe sepsis w mv >96 hours or peripheral extracorporeal membrane oxygenation (ecmo), 871 Septicemia or severe sepsis w/o mv >96 hours w mcc, 872 Septicemia or severe sepsis w/o mv >96 hours w/o mcc)
COPD Exacerbation	((A AND B AND C AND D) OR (E AND F AND G) OR (H AND I AND J AND K)) AND L, where<br>A = Principal Diagnosis ICD-10(j441)<br>B = Any Diagnosis ICD-10(j9601, j9602, j9621, j9622)<br>C = Any Diagnosis ICD-10(i5032, e440)<br>D = Any Diagnosis ICD-10(E872, E8720, E8729, E873)<br>E = Principal Diagnosis ICD-10(j9621)<br>F = Any Diagnosis ICD-10(j441)<br>G = Any Diagnosis ICD-10(I5020, I5021, I5022, I5023, I503, I5030, I5031, I5033, I5041, I5043)<br>H = Principal Diagnosis ICD-10(j440)<br>I = Any Diagnosis ICD-10(j441)<br>J = Any Diagnosis ICD-10(j189)<br>K = Any Diagnosis ICD-10(n179)<br>L = Any Diagnosis Not ICD-10(r570)
Bacterial Pneumonia	(A AND B) AND C AND (D AND E) AND F, where:<br>A = MS-DRG (177 Respiratory infections & inflammations w mcc, 178 Respiratory infections & inflammations w cc, 179 Respiratory infections & inflammations w/o cc/mcc, 189 Pulmonary edema & respiratory failure, 193 Simple pneumonia & pleurisy w mcc, 194 Simple pneumonia & pleurisy w cc, 195 Simple pneumonia & pleurisy w/o cc/mcc, 871 Septicemia or severe sepsis w/o mv >96 hours w mcc, 872 Septicemia or severe sepsis w/o mv >96 hours w/o mcc, 974 Hiv w major related condition w mcc, 975 Hiv w major related condition w cc, 976 Hiv w major related condition w/o cc/mcc)<br>B = Any Diagnosis ICD-10(J13, J14, J15, J150, J151, J152, J1520, J1521, J15211, J15212, J1529, J153, J154, J155, J156, J157, J158, J159, J18, J180, J181, J189, J188, J690)<br>C = Any Diagnosis Not ICD-10(A045, A0472, A150, A159, A199, A310, A319, A539, B3781, B382, B451, B9719, B9781, C187, C3490, C469, C679, C7951, C8510, C8589, C8591, C9110, C9150, C9200, C9500, D471, D47Z2, D5700, D57459, D6861, E035, E213, E222, E883, F0150, F1590, F209, F842, F99, G061,

© 2024 Banerjee J et al. *JAMA Network Open.*

	G3183, G40812, G450, G7100, G800, G803, G809, G928, G931, G9529, I213, I214, I219, I2693, I2699, I2721, I3139, I330, I350, I351, I452, I469, I471, I499, I6340, I63411, I639, I6602, I69344, I69351, I69354, J1000, J1008, J1282, J471, J479, J80, J869, J939, J942, J95851, J9622, K222, K224, K5130, K5289, K529, K55069, K5530, K5790, K625, K7200, K82A2, K8309, K831, K9049, L02211, L958, M3130, M3131, M479, N809, R419, R45851, R570, R571, R6510, S025XXA, S065X0A, S065XAA, S24104S, S32511A, T368X5A, T40411A, Z515, Z8661, Z87820, Z882, Z90710, Z9989)<br>D = Any Diagnosis Not ICD-10(U071)<br>E = Any Diagnosis Not ICD-10(J2699)<br>F = Any Procedure Not ICD-10(0bh17ez, 0bh18ez)
CHF	((A AND B AND C AND D) OR (E AND B AND F) OR (G AND B AND H AND I) OR (J AND K AND L) OR (M AND B AND N AND O)) AND P AND Q, where:<br>A = Principal Diagnosis ICD-10(I110)<br>B = Any Diagnosis ICD-10(I5023, I5033, I5021, I5031, I5041, I5043)<br>C = Any Diagnosis ICD-10(j9601, j9611, i2510)<br>D = Any Diagnosis ICD-10(I2720, I2729, I26, I269, I2693, I2694, I2699, J189, I480, I481, I4891, I429, I427, I426, I420)<br>E = Principal Diagnosis ICD-10(i2510)<br>F = Any Diagnosis ICD-10(j9601, i21a1, i25, i130, n1830)<br>G = Principal Diagnosis ICD-10(I13, I130, I131, I1310, I1311, I132)<br>H = Any Diagnosis ICD-10(i21a1, i2720, n179, j9601, j9612, i2510)<br>I = Any Diagnosis ICD-10(i48, i31, n189, n1831, n182, n186)<br>J = Principal Diagnosis ICD-10(I5023, I5033, I5021, I5031, I5041, I5043)<br>K = Any Diagnosis ICD-10(i3139, e440)<br>L = Any Diagnosis ICD-10(i351, f1010)<br>M = Principal Diagnosis ICD-10(e11649, r0789)<br>N = Any Diagnosis ICD-10(I110, I13, I130, I131, I1310, I1311, I132)<br>O = Any Diagnosis ICD-10(k7200, i428, i48, e119, i2720, i2510)<br>P = Any Diagnosis Not ICD-10(i462, i469, j1282, m86172, s22049a)<br>Q = Any Procedure Not ICD-10(5a02210, 5a1955z, 0jh608z, 0ybk0zz, 027034z, 06b43zx, 0jh634z, 0b110f4, 02rf38z, 0b9d8zx, 027236z, 027135z)
Asthma/Bronchitis	((A AND B) OR (A AND C AND D) OR (A AND (K AND L)) OR (E AND F) OR (G AND H AND I)) AND J, where<br>A = Principal Diagnosis ICD-10(J40, J4541, J4542, J4551, J4552, J45901, J45909)<br>B = Any Diagnosis ICD-10(B9689, J4542, J9600, J9601, J9602, R0902, T782XXA, T783XXA)<br>C = Any Diagnosis ICD-10(Z20822)

© 2024 Banerjee J et al. *JAMA Network Open.*

	D = Any Diagnosis ICD-10(Z79899)<br>E = Principal Diagnosis ICD-10(J9600, J9601, J9602, O99512)<br>F = Secondary Diagnosis ICD-10(J4541, J4542, J4551, J4552, J45901, J45909)<br>G = Principal Diagnosis ICD-10(J069)<br>H = Secondary Diagnosis ICD-10(J4541, J4542, J4551, J45901)<br>I = Secondary Diagnosis ICD-10(J9600, J9601, J9602)<br>J = Any Procedure Not ICD-10(0BH17EZ, 0BH18EZ, 3E033XZ)<br>K = Secondary Diagnosis ICD-10(R0602, R0609)<br>L = Secondary Diagnosis ICD-10(R000)
COVID Pneumonia	((A AND B) OR (C OR D) OR (E AND F AND G)) AND H AND I where:<br>A = Any Diagnosis ICD-10(J1282)<br>B = Any Diagnosis ICD-10(J9601)<br>C = Principal Diagnosis ICD-10(U071)<br>D = Secondary Diagnosis ICD-10(J1282)<br>E = Principal Diagnosis ICD-10(o98513, i6389)<br>F = Secondary Diagnosis ICD-10(U071)<br>G = Secondary Diagnosis ICD-10(r000, i6522)<br>H = Any Procedure Not ICD-10(0bh17ez, 0bh18ez, 3E033XZ)<br>I = Any Diagnosis Not ICD-10(0ft44zz, k710, k750, i81, k7200, i493, t7401xa, k551, f849, i21a1, l02416, i69354, f842, i469, s2242xa, i5033, q210, k651, d47z2, z6843, i69954, i5022, i248, c7951, i2720, m3214, r45851, a4159, i214, k658, o368330, e883, b387, r0789, i7782, f15259, b974, i69359, r571, r9431, d5700, i471, c9000, j050, i2119, o6003, a084, i340, s72002a, b59, n390, i513, j218, n130, i69951, b451, j069, m6282, n939, j690, i6389, i2699, c8590, f0390, r339, p7431, d61810, r061, z940, q315, e43, i495, c7800, i4891, z950, z1612, e0500, s01111a, b349, r040, j029, k9049, r5600, i161, i69322, p221, j45901, a319, j45902, g936, q2110, i6523, e872, e8721, r55, i318)
Acute Respiratory Failure Due to Other Viruses	((A AND B AND C AND D) OR (E AND F AND G) OR (H AND I AND J) OR (K AND L AND M)) AND N, where:<br>A = Principal Diagnosis ICD-10(a419)<br>B = Any Diagnosis ICD-10(J12, J128, J123, J122, J121, J120, J1289, J129, J100, J1000, J1001, J1008)<br>C = Any Diagnosis ICD-10(E871, J960, J9600, J9601)<br>D = Any Diagnosis ICD-10(E861, N17, N170, N171, N172, N178, N179)<br>E = Principal Diagnosis ICD-10(J1000, J1008, J069)<br>F = Any Diagnosis ICD-10(J960, J9600, J9601)<br>G = Any Diagnosis ICD-10(E110, E1100, E1110, E112, E1121, E1122, E1129, E113, E1131, E11311, E11319, E1132, E1169, E1165, E118, E119)<br>H = Principal Diagnosis ICD-10(J121)<br>I = Any Diagnosis ICD-10(i110)<br>J = Any Diagnosis ICD-10(I5031, I5033)<br>K = Principal Diagnosis ICD-10(J069)<br>L = Any Diagnosis ICD-10(B9789)

© 2024 Banerjee J et al. *JAMA Network Open.*

	M = Any Diagnosis ICD-10(j9600, j9601)<br>N = Any Diagnosis Not ICD-10(r6521, u071, z89422, z8719, g40812, b9719, p2849, i63232)
Gout	Principal Diagnosis ICD-10(M109) AND Any Diagnosis Not ICD-10(i38)
Bacterial Prostatitis	Principal Diagnosis ICD-10(N410)
Pneumocystis Pneumonia	Principal Diagnosis ICD-10(B20) AND Any Diagnosis ICD-10(B59) AND Any Diagnosis ICD-10(J9601) AND Any Diagnosis Not ICD-10(j449, i469)
Seizures Due to External Causes	Any Diagnosis ICD-10(g40509) AND Any Diagnosis ICD-10(r509) AND Any Diagnosis ICD-10(F10, F101, F1010, F1011, F1012, F10120, F10121, F10129, F10130, F10131, F10132, F10139, F1014, F1015, F10150, F10151, F10159, F1018, F10180, F10181, F10182, F10188, F1019, F102, F1020, F1021, F1022, F10220, F10221, F10229, F1023, F10230, F10231, F10232, F10239, F1024, F1025, F10250, F10251, F10259, F1026, F1027, F1028, F10280, F10281, F10282, F10288, F1029, F109, F1090, F1091, F1092, F10920, F10921, F10929, F10930, F10931, F10932, F10939, F1094, F1095, F10950, F10951, F10959, F1096, F1097, F1098, F10980, F10981, F10982, F10988, F1099, G312, G621, G721, I426, K292, K2920, K2921, K70, K700, K701, K7010, K7011, K702, K703, K7030, K7031, K704, K7040, K7041, K709, K7581, K852, K8520, K8521, K8522, K860, O354, O354XX0, O354XX1, O354XX2, O354XX3, O354XX4, O354XX5, O354XX9, O9931, O99310, O99311, O99312, O99313, O99314, O99315, P043, Q860, R780, T51, T518, T518X, T518X1, T518X1A, T518X1D, T518X1S, T518X2, T518X2A, T518X2D, T518X2S, T518X3, T518X3A, T518X3D, T518X3S, T518X4, T518X4A, T518X4D, T518X4S, T519, T5191, T5191XA, T5191XD, T5191XS, T5192, T5192XA, T5192XD, T5192XS, T5193, T5193XA, T5193XD, T5193XS, T5194, T5194XA, T5194XD, T5194XS, Y90, Y900, Y901, Y902, Y903, Y904, Y905, Y906, Y907, Y908, Y909, Z0283, Z6372, Z714, Z7141, Z7142, Z811, E244)
S. *aureus* Sepsis/Bacteremia	Any Diagnosis ICD-10(A410, A4101, A4102, A4901, A4902) AND Any Diagnosis ICD-10(R6521, I33, I330, I339, I38, I39, J152, J1520, J1521, J15211, J15212, J1529, M462, M4620, M4622, M4623, M4624, M4625, M4627, M4626, M4628, H0502, H05021, H05022, H05023, H05029, M4621, M86, M860, M8600, M8601, M86011, M86012, M86019, M8602, M86021, M86022, M86029, M8603, M86031, M86032, M86039, M8604, M86041, M86042, M86049, M8605, M86051, M86052, M86059, M8606, M86061, M86062, M86069, M8607, M86071, M86072, M86079, M8608, M8609, M861, M8610, M8611, M86111, M86112, M86119, M8612, M86121, M86122, M86129, M8613, M86131, M86132, M86139, M8614, M86141, M86142, M86149, M8615, M86151, M86152, M86159, M8616, M86161, M86162, M86169, M8617, M86171, M86172, M86179, M8618, M8619, M862, M8620,

© 2024 Banerjee J et al. *JAMA Network Open.*

	M8621, M86211, M86212, M86219, M8622, M86221, M86222, M86229, M8623, M86231, M86232, M86239, M8624, M86241, M86242, M86249, M8625, M86251, M86252, M86259, M8626, M86261, M86262, M86269, M8627, M86271, M86272, M86279, M8628, M8629, M863, M8630, M8631, M86311, M86312, M86319, M8632, M86321, M86322, M86329, M8633, M86331, M86332, M86339, M8634, M86341, M86342, M86349, M8635, M86351, M86352, M86359, M8636, M86361, M86362, M86369, M8637, M86371, M86372, M86379, M8638, M8639, M864, M8640, M8641, M86411, M86412, M86419, M8642, M86421, M86422, M86429, M8643, M86431, M86432, M86439, M8644, M86441, M86442, M86449, M8645, M86451, M86452, M86459, M8646, M86461, M86462, M86469, M8647, M86471, M86472, M86479, M8648, M8649, M865, M8650, M8651, M86511, M86512, M86519, M8652, M86521, M86522, M86529, M8653, M86531, M86532, M86539, M8654, M86541, M86542, M86549, M8655, M86551, M86552, M86559, M8656, M86561, M86562, M86569, M8657, M86571, M86572, M86579, M8658, M8659, M866, M8660, M8661, M86611, M86612, M86619, M8662, M86621, M86622, M86629, M8663, M86631, M86632, M86639, M8664, M86641, M86642, M86649, M8665, M86651, M86652, M86659, M8666, M86661, M86662, M86669, M8667, M86671, M86672, M86679, M8668, M8669, M868, M868X, M868X0, M868X1, M868X2, M868X3, M868X4, M868X5, M868X6, M868X7, M868X8, M868X9, M869, a419) AND Any Diagnosis Not ICD-10(a4151, z515, r402112, f0390, i676, g062, j1282, i82413, a408, j155, a403, t2026xa) AND Any Procedure Not ICD-10(00c40zz, 009630z, 03c84zz, 4a023n6, 5a1955z, 02cj3zz, 02rj08z, 0rg20j1, 02rj0jz, 02ch3zz, 0sbc0zz, 08943zz, 02qv0zz, 047l3z1, 0x6r0z2, 0qb00zz, 041l09m, 0y6j0z1, 0pb60zz, 0x6m0z3)
Severe Sepsis/Shock with Respiratory Failure	Principal Diagnosis ICD-10(A40, A400, A401, A403, A408, A409, A41, A413, A415, A4150, A4151, A4152, A4153, A4159, A418, A419) AND Any Diagnosis ICD-10(j9601) AND Any Diagnosis ICD-10(r6520, r6521) AND Any Procedure ICD-10(5a1945z) AND Any Procedure Not ICD-10(5a0955a, 5a1955z, 02ha3rz) AND Any Diagnosis Not ICD-10(g360, i469, z515, g231, t8641, i720, i6203, i493, k7200, g931, j9602, i071, s0219xa, i6359, t40414a, t402x1a, r402342, k831, t68xxxa, b965, z8673)
ESBL Bacteremia	Any Diagnosis ICD-10(Z1612) AND Any Diagnosis ICD-10(R7881) AND Any Diagnosis Not ICD-10(z651, i469, 4a103bd, a312, k767, e883, g936, g389, g308, g061, e279, j80, f209, i69951, C228, z85028, d531, c7931, c8589, r402212, r402342, r402432, e2740, e035, s06a1xa, i81, y92009, k7201, i7409, k55059, s7221xa, g9382) AND Any Procedure Not ICD-10(5a02210, 5a1d90z, 5a0955a, 5a0955z, 5a12012, 0w3g0zz, 4b02xsz, 0b9j8zx, 3E033XZ, 0bh17ez, 0bh18ez)
HD Catheter Bacteremia	Principal Diagnosis ICD-10(A410, A41, A4101, A4102, A411, A412, A415, A4150, A4151, A4152, A4153, A4159, A418, A4181,

© 2024 Banerjee J et al. *JAMA Network Open.*

	A4189, A419) AND Any Procedure Not ICD-10(05wy4jz) AND Any Procedure ICD-10(0jpt3xz)
Epididymo-Orchitis	Principal Diagnosis ICD-10(A41, A415, A4150, A4151, A4152, A4153, A4159, A418, A4181, A4189, A419) AND Any Diagnosis ICD-10(N45, N451, N452, N453)
Complicated Intra-Abdominal Infection	((A AND B) OR (C AND D) OR (E AND F) OR (G AND H) OR (I AND J)) AND K AND L where:<br>A = Any Diagnosis ICD-10(k750)<br>B = Any Procedure ICD-10(0f9030z, 0fb13zx)<br>C = Any Diagnosis ICD-10(k8590)<br>D = Any Diagnosis ICD-10(k5732, f10230)<br>E = Any Diagnosis ICD-10(a049)<br>F = Any Diagnosis ICD-10(n170)<br>G = Any Diagnosis ICD-10(k3532)<br>H = Any Diagnosis ICD-10(i959)<br>I = Any Diagnosis ICD-10(s31630a)<br>J = Any Diagnosis ICD-10(t794xxa, s36116a, k661)<br>K = Any Diagnosis Not ICD-10(r6521, c220, g540, s82391b, s72114a, i21a1, s65011a)<br>L = Any Procedure Not ICD-10(02qa0zz)
Alcoholic Cirrhosis	Any Diagnosis ICD-10(e8779) AND Any Diagnosis ICD-10(K7031) AND Any Procedure ICD-10(0w9g3zz)
Acute Hypoxic Respiratory Failure	((A AND B AND C) OR (A AND D AND E) OR ((A OR F OR K) AND G AND B) OR (A AND I AND K) OR (K AND (A OR F OR J))) AND H, where:<br>A = Any Diagnosis ICD-10(J9601)<br>B = Any Diagnosis ICD-10(I21a1)<br>C = Any Diagnosis ICD-10(J189)<br>D = Any Procedure ICD-10(0w993zz)<br>E = Any Diagnosis ICD-10(r0902, C384, j910)<br>F = Any Diagnosis ICD-10(r0902, C384, j910)<br>G = Any Diagnosis ICD-10(e662)<br>H = Any Diagnosis Not ICD-10(I468, G039, r570, k7682, k201, n12, i5030, i5032, z515, j1282, i330, k8510, i63442, g08, b59, f15188, z9989, r578, z91128, t83511a, i080, i63531, i69351, c787, t402x1a, c779, e883, c9200, i69954, g9601, g928, f1399, i97130, z952, m8668, k5792, j129, e43, f0280, k863, z8673, i314, a0472, z955)<br>I = Any Procedure ICD-10(5a09357, 5a0945a)<br>J = Encounter Number Not(1032242033, 1031497363, 1031642578, 1031832839, 1031895981, 1031971991, 1031924096, 1032149858, 1033290419, 1034404945, 1034631480)<br>K = Principal Diagnosis ICD-10(i2699)
Rhabdomyolysis with Acute Kidney Injury	((A AND B AND C) OR (D AND E AND F)) AND G, where:<br>A = Principal Diagnosis ICD-10(m6282)<br>B = Any Diagnosis ICD-10(N17, N170, N171, N172, N178, N179)<br>C = Any Diagnosis ICD-10(R7401)<br>D = Any Diagnosis ICD-10(m6282)

© 2024 Banerjee J et al. *JAMA Network Open.*

	E = Any Diagnosis ICD-10(k7200)<br>F = Any Diagnosis ICD-10(i21a1)<br>G = Any Diagnosis Not ICD-10(i618, g936, r6521, f05, s36031a)
Gastroenteritis	Principal Diagnosis ICD-10(A09) AND Any Diagnosis Not ICD-10(j189, t8452xa)
Diverticulitis	Principal Diagnosis ICD-10(K5793, K5732, K5712) AND Any Diagnosis Not ICD-10(n739, c786, i69351, i471, z651, g9340, e7401) AND Any Procedure Not ICD-10(0dbm0zz, 0dbn4zz, 0dtn0zz, 0dbn4zx, 5a19054, 0dtn4zz, 0dbn0zz, 0dbn0zx)
Cholangitis with Bacteremia	Any Diagnosis ICD-10(A4151, A4153) AND Any Diagnosis ICD-10(K800, K8001, K804, K8041, K8043, k8041, k8063)
Acute Viral Hepatitis	Principal Diagnosis ICD-10(B16, B169) AND Any Diagnosis ICD-10(r7401)
Cancer Related Cauda Equina	Any Diagnosis ICD-10(G834) AND Any Diagnosis Not ICD-10(z651, i469, 4a103bd, a312, k767, e883, g936, g389, g308, g061, e279, j80, f209, i69951, C228, z85028, d531, c7931, c8589, r402212, r402342, r402432, e2740, e035, s06a1xa, i81, y92009, k7201, i7409, k55059, s7221xa, g9382, i21a1, n390, m4626, m4628, g600, k651, e724, g9519, m868x8, f10939, U071, k5289, j340, f10239) AND Any Procedure Not ICD-10(5a02210, 5a1d90z, 5a0955a, 5a0955z, 5a12012, 0w3g0zz, 4b02xsz, 0b9j8zx, 00nx0zz, 0gtg0zz, 08sl3zz, 00bc0zz, 0sb20zz, 0rg10j1, 0sg10aj, 00ny0zz, 01nb0zz, 01nb0zz, g40909, 0psf04z, 0sg1071, 0sg10aj, 00bt0zx, 0rg2071, 01nb3zz)
Metastatic Cancer with Necrotizing Fasciitis	Any Diagnosis ICD-10(c779) AND Any Diagnosis ICD-10(m726)
Neutropenic Fever	Any Diagnosis ICD-10(R509, R5081, R50, R508, R5084) AND Any Diagnosis ICD-10(D70, D701, D708, D709) AND Any Diagnosis ICD-10(C9200) AND Any Diagnosis Not ICD-10(k651, e883, u071, 9601, k9049, k1230, r6521, k810) AND Any Procedure Not ICD-10(0u9l0zz)
Fever Without a Source	Principal Diagnosis ICD-10(R50, R508, R509)
Acute Kidney Injury	(A OR (B AND C)) AND D AND E, where:<br>A = Principal Diagnosis ICD-10(n170)<br>B = Principal Diagnosis ICD-10(e1121)<br>C = Any Diagnosis ICD-10(n179)<br>D = Any Diagnosis Not ICD-10(i81, k767, u071, n059, t8611, K7200, c73, j9601, i32, i6381, e878, i959, h579)<br>E = Any Procedure Not ICD-10(04ld3dz, 0tb13zx)
Pulmonary Embolus	Principal Diagnosis ICD-10(I269, I2699) AND Any Diagnosis ICD-10(J459, J4590, J45909, J4599) AND Any Diagnosis Not ICD-10(J9601)

© 2024 Banerjee J et al. *JAMA Network Open.*

eTable 3. Definition of Cases of Cellulitis, Diabetic Foot Infection, and Pyelonephritis Presenting to Emergency Department (ED), by ICD-10-CM Codes

Diagnosis	ICD-10-CM Codes
Cellulitis	Any Dx 1-5 on ED encounter: L03(Cellulitis and acute lymphangitis); L03.0(Cellulitis and acute lymphangitis of finger and toe); L03.01(Cellulitis of finger); L03.011(Cellulitis of right finger); L03.012(Cellulitis of left finger); L03.019(Cellulitis of unspecified finger); L03.02(Acute lymphangitis of finger); L03.021(Acute lymphangitis of right finger); L03.022(Acute lymphangitis of left finger); L03.029(Acute lymphangitis of unspecified finger); L03.03(Cellulitis of toe); L03.031(Cellulitis of right toe); L03.032(Cellulitis of left toe); L03.039(Cellulitis of unspecified toe); L03.04(Acute lymphangitis of toe); L03.041(Acute lymphangitis of right toe); L03.042(Acute lymphangitis of left toe); L03.049(Acute lymphangitis of unspecified toe); L03.1(Cellulitis and acute lymphangitis of other parts of limb); L03.11(Cellulitis of other parts of limb); L03.111(Cellulitis of right axilla); L03.112(Cellulitis of left axilla); L03.113(Cellulitis of right upper limb); L03.114(Cellulitis of left upper limb); L03.115(Cellulitis of right lower limb); L03.116(Cellulitis of left lower limb); L03.119(Cellulitis of unspecified part of limb); L03.12(Acute lymphangitis of other parts of limb); L03.121(Acute lymphangitis of right axilla); L03.122(Acute lymphangitis of left axilla); L03.123(Acute lymphangitis of right upper limb); L03.124(Acute lymphangitis of left upper limb); L03.125(Acute lymphangitis of right lower limb); L03.126(Acute lymphangitis of left lower limb); L03.129(Acute lymphangitis of unspecified part of limb); L03.2(Cellulitis and acute lymphangitis of face and neck); L03.21(Cellulitis and acute lymphangitis of face); L03.211(Cellulitis of face); L03.212(Acute lymphangitis of face); L03.213(Periorbital cellulitis); L03.22(Cellulitis and acute lymphangitis of neck); L03.221(Cellulitis of neck); L03.222(Acute lymphangitis of neck); L03.3(Cellulitis and acute lymphangitis of trunk); L03.31(Cellulitis of trunk); L03.311(Cellulitis of abdominal wall); L03.312(Cellulitis of back [any part except buttock]); L03.313(Cellulitis of chest wall); L03.314(Cellulitis of groin); L03.315(Cellulitis of perineum); L03.316(Cellulitis of umbilicus);

© 2024 Banerjee J et al. *JAMA Network Open.*

	L03.317(Cellulitis of buttock); L03.319(…… unspecified); L03.32(Acute lymphangitis of trunk); L03.321(Acute lymphangitis of abdominal wall); L03.322(Acute lymphangitis of back [any part except buttock]); L03.323(Acute lymphangitis of chest wall); L03.324(Acute lymphangitis of groin); L03.325(Acute lymphangitis of perineum); L03.326(Acute lymphangitis of umbilicus); L03.327(Acute lymphangitis of buttock); L03.329(…… unspecified); L03.8(Cellulitis and acute lymphangitis of other sites); L03.81(Cellulitis of other sites); L03.811(Cellulitis of head [any part, except face]); L03.818(Cellulitis of other sites); L03.89(Acute lymphangitis of other sites); L03.891(Acute lymphangitis of head [any part, except face]); L03.898(Acute lymphangitis of other sites); L03.9(Cellulitis and acute lymphangitis, unspecified); L03.90(Cellulitis, unspecified); L03.91(Acute lymphangitis, unspecified); H05.01(Cellulitis of orbit); H05.011(Cellulitis of right orbit); H05.012(Cellulitis of left orbit); H05.013(Cellulitis of bilateral orbits); H05.019(Cellulitis of unspecified orbit); L02(Cutaneous abscess, furuncle and carbuncle); L02.0(Cutaneous abscess, furuncle and carbuncle of face); L02.01(Cutaneous abscess of face); L02.02(Furuncle of face); L02.03(Carbuncle of face); L02.1(Cutaneous abscess, furuncle and carbuncle of neck); L02.11(Cutaneous abscess of neck); L02.12(Furuncle of neck); L02.13(Carbuncle of neck); L02.2(Cutaneous abscess, furuncle and carbuncle of trunk); L02.21(Cutaneous abscess of trunk); L02.211(Cutaneous abscess of abdominal wall); L02.212(Cutaneous abscess of back [any part, except buttock]); L02.213(Cutaneous abscess of chest wall); L02.214(Cutaneous abscess of groin); L02.215(Cutaneous abscess of perineum); L02.216(Cutaneous abscess of umbilicus); L02.219(…… unspecified); L02.22(Furuncle of trunk); L02.221(Furuncle of abdominal wall); L02.222(Furuncle of back [any part, except buttock]); L02.223(Furuncle of chest wall); L02.224(Furuncle of groin); L02.225(Furuncle of perineum); L02.226(Furuncle of umbilicus); L02.229(…… unspecified); L02.23(Carbuncle of trunk); L02.231(Carbuncle of abdominal wall); L02.232(Carbuncle of back [any part, except buttock]); L02.233(Carbuncle of chest wall); L02.234(Carbuncle of groin); L02.235(Carbuncle of perineum); L02.236(Carbuncle of umbilicus);

© 2024 Banerjee J et al. *JAMA Network Open.*

	L02.239(…… unspecified); L02.3(Cutaneous abscess, furuncle and carbuncle of buttock); L02.31(Cutaneous abscess of buttock); L02.32(Furuncle of buttock); L02.33(Carbuncle of buttock); L02.4(Cutaneous abscess, furuncle and carbuncle of limb); L02.41(Cutaneous abscess of limb); L02.411(Cutaneous abscess of right axilla); L02.412(Cutaneous abscess of left axilla); L02.413(Cutaneous abscess of right upper limb); L02.414(Cutaneous abscess of left upper limb); L02.415(Cutaneous abscess of right lower limb); L02.416(Cutaneous abscess of left lower limb); L02.419(…… unspecified); L02.42(Furuncle of limb); L02.421(Furuncle of right axilla); L02.422(Furuncle of left axilla); L02.423(Furuncle of right upper limb); L02.424(Furuncle of left upper limb); L02.425(Furuncle of right lower limb); L02.426(Furuncle of left lower limb); L02.429(…… unspecified); L02.43(Carbuncle of limb); L02.431(Carbuncle of right axilla); L02.432(Carbuncle of left axilla); L02.433(Carbuncle of right upper limb); L02.434(Carbuncle of left upper limb); L02.435(Carbuncle of right lower limb); L02.436(Carbuncle of left lower limb); L02.439(…… unspecified); L02.5(Cutaneous abscess, furuncle and carbuncle of hand); L02.51(Cutaneous abscess of hand); L02.511(Cutaneous abscess of right hand); L02.512(Cutaneous abscess of left hand); L02.519(Cutaneous abscess of unspecified hand); L02.52(Furuncle hand); L02.521(Furuncle right hand); L02.522(Furuncle left hand); L02.529(Furuncle unspecified hand); L02.53(Carbuncle of hand); L02.531(Carbuncle of right hand); L02.532(Carbuncle of left hand); L02.539(Carbuncle of unspecified hand); L02.6(Cutaneous abscess, furuncle and carbuncle of foot); L02.61(Cutaneous abscess of foot); L02.611(Cutaneous abscess of right foot); L02.612(Cutaneous abscess of left foot); L02.619(Cutaneous abscess of unspecified foot); L02.62(Furuncle of foot); L02.621(Furuncle of right foot); L02.622(Furuncle of left foot); L02.629(Furuncle of unspecified foot); L02.63(Carbuncle of foot); L02.631(Carbuncle of right foot); L02.632(Carbuncle of left foot); L02.639(Carbuncle of unspecified foot); L02.8(Cutaneous abscess, furuncle and carbuncle of other sites); L02.81(Cutaneous abscess of other sites); L02.811(Cutaneous abscess of head [any part, except face]); L02.818(Cutaneous abscess of

© 2024 Banerjee J et al. *JAMA Network Open.*

	other sites); L02.82(Furuncle of other sites); L02.821(Furuncle of head [any part, except face]); L02.828(Furuncle of other sites); L02.83(Carbuncle of other sites); L02.831(Carbuncle of head [any part, except face]); L02.838(Carbuncle of other sites); L02.9(Cutaneous abscess, furuncle and carbuncle, unspecified); L02.91(Cutaneous abscess, unspecified); L02.92(Furuncle, unspecified); L02.93(Carbuncle, unspecified)
Diabetic Foot Infection	Any Dx 1-5 on ED encounter: E08.62(Diabetes mellitus due to underlying condition with skin complications); E08.620(Diabetes mellitus due to underlying condition with diabetic dermatitis); E08.621(Diabetes mellitus due to underlying condition with foot ulcer); E08.622(Diabetes mellitus due to underlying condition with other skin ulcer); E08.628(Diabetes mellitus due to underlying condition with other skin complications); E09.62(Drug or chemical induced diabetes mellitus with skin complications); E09.620(Drug or chemical induced diabetes mellitus with diabetic dermatitis); E09.621(Drug or chemical induced diabetes mellitus with foot ulcer); E09.622(Drug or chemical induced diabetes mellitus with other skin ulcer); E09.628(Drug or chemical induced diabetes mellitus with other skin complications); E10.62(Type 1 diabetes mellitus with skin complications); E10.620(Type 1 diabetes mellitus with diabetic dermatitis); E10.621(Type 1 diabetes mellitus with foot ulcer); E10.622(Type 1 diabetes mellitus with other skin ulcer); E10.628(Type 1 diabetes mellitus with other skin complications); E11.62(Type 2 diabetes mellitus with skin complications); E11.620(Type 2 diabetes mellitus with diabetic dermatitis); E11.621(Type 2 diabetes mellitus with foot ulcer); E11.622(Type 2 diabetes mellitus with other skin ulcer); E11.628(Type 2 diabetes mellitus with other skin complications); E13.62(Other specified diabetes mellitus with skin complications); E13.620(Other specified diabetes mellitus with diabetic dermatitis); E13.621(Other specified diabetes mellitus with foot ulcer); E13.622(Other specified diabetes mellitus with other skin ulcer); E13.628(Other specified diabetes mellitus with other skin complications)
Pyelonephritis	Any Dx 1-5 on ED encounter: N10(Acute pyelonephritis); N11.0(Nonobstructive reflux-associated chronic pyelonephritis); N11.1(Chronic

© 2024 Banerjee J et al. *JAMA Network Open.*

	obstructive pyelonephritis); N11.8(Other chronic tubulo-interstitial nephritis); N11.9(Chronic tubulo-interstitial nephritis, unspecified); N12(Tubulo-interstitial nephritis, not specified as acute or chronic); N39.0(Urinary tract infection, site not specified); N13.6(Pyonephrosis); N15.1(Renal and perinephric abscess); N28.85(Pyeloureteritis cystica); N30.00(Acute cystitis without hematuria); N30.01(Acute cystitis with hematuria)

© 2024 Banerjee J et al. *JAMA Network Open.*

eTable 4. Characteristics of Patients With "Core" Diagnoses

Characteristic	Safer@Home Cohort	Control Cohort	P Value
Care Setting at Discharge--no.(%)			
Hospital	346 (42%)	1354 (100%)	
Emergency Department / Observation / Urgent Care	477 (58%)	0 (0%)	
Total	823	1354	
Mean (SD) Age (years)	53.7 (16.1)	52.6 (20)	0.31
Sex--no.(%)			0.15
Female	172 (49.7%)	622 (45.9%)	
Male	175 (50.6%)	732 (54.1%)	
Race/Ethnicity--no.(%)			0.25
Asian	17 (1.3%)	66 (4.9%)	
Black	11 (3.2%)	79 (5.8%)	
Hispanic	287 (83%)	1067 (79%)	
Other	22 (6.4%)	93 (6.9%)	
White	10 (2.9%)	49 (3.6%)	
Mean (SD) Case Mix Index	1.19 (0.497)	1.19 (0.45)	0.83
Mean (SD) Expected Mortality	0.0115 (0.0363)	0.0144 (0.0415)	0.30
Mean (SD) Length of Stay	1.12 (1.38)	4.62 (9.09)	<0.001

© 2024 Banerjee J et al. *JAMA Network Open.*

eTable 5. Characteristics of Patients With Other Diagnoses

Characteristic	Safer@Home Cohort	Control Cohort	P Value
Care Setting at Discharge--no.(%)			
Hospital	47 (88.7%)	236 (100%)	
Emergency Department / Observation / Urgent Care	6 (11.3%)	0 (0%)	
Total	53	236	
Mean (SD) Age (years)	54.7 (15.7)	50.8 (16.7)	0.14
Sex--no.(%)			0.91
Female	14 (29.8%)	69 (29.2%)	
Male	34 (72.3%)	167 (70.8%)	
Race/Ethnicity--no.(%)			0.43
Asian	2 (4.3%)	9 (3.8%)	
Black	3 (6.4%)	29 (12.3%)	
Hispanic	39 (83%)	168 (71.2%)	
Other	4 (8.5%)	20 (8.5%)	
White	0 (0%)	10 (4.2%)	
Mean (SD) Case Mix Index	1.85 (1.19)	1.62 (1.03)	0.17
Mean (SD) Expected Mortality	0.0476 (0.0858)	0.0433 (0.114)	0.80
Mean (SD) Length of Stay	4.54 (5.05)	9.46 (15.55)	0.02

© 2024 Banerjee J et al. *JAMA Network Open.*

eTable 6. Characteristics of Patients With Cellulitis

Characteristic	Safer@Home Cohort	Control Cohort	P Value
Care Setting at Discharge--no.(%)			
Hospital	58 (46%)	304 (100%)	
Emergency Department / Observation / Urgent Care	68 (54%)	0 (0%)	
Total	126	304	
Mean (SD) Age (years)	49.4 (14.9)	47.9 (15.2)	0.44
Sex--no.(%)			0.77
Female	21 (36.2%)	104 (34.2%)	
Male	37 (63.8%)	200 (65.8%)	
Race/Ethnicity--no.(%)			0.69
Asian	4 (6.9%)	9 (3.0%)	
Black	1 (1.7%)	7 (2.3%)	
Hispanic	43 (74.1%)	234 (77%)	
White	5 (8.6%)	28 (9.2%)	
Other*	5 (8.6%)	26 (8.6%)	
Mean (SD) Case Mix Index	1.13 (0.592)	1.05 (0.451)	0.64
Mean (SD) Expected Mortality	0.00593 (0.00998)	0.00668 (0.0179)	0.26
Mean (SD) Length of Stay	1.059 (0.87)	4.36 (7.49)	<0.001

*Other was based on patient preference as entered by the nurse in the electronic medical record.

© 2024 Banerjee J et al. *JAMA Network Open.*

eTable 7. Characteristics of Patients With Diabetic Foot Infections

Characteristic	Safer@Home Cohort	Control Cohort	P Value
Care Setting at Discharge--no.(%)			
Hospital	66 (22.1%)	120 (100%)	
Emergency Department / Observation / Urgent Care	233 (77.9%)	0 (0%)	
Total	299	120	
Mean (SD) Age (years)	52.2 (10.40)	54.7 (9.97)	0.10
Sex--no.(%)			0.54
Female	14 (21.2%)	21 (17.5%)	
Male	52 (78.8%)	99 (82.5%)	
Race/Ethnicity--no.(%)			0.17
Asian	0 (0%)	3 (2.5%)	
Black	0 (0%)	7 (5.8%)	
Hispanic	65 (98.5%)	106 (88.3%)	
White	0 (0%)	0 (0%)	
Other*	1 (1.5%)	4 (3.3%)	
Mean (SD) Case Mix Index	1.06 (0.34)	1.11 (0.55)	0.53
Mean (SD) Expected Mortality	0.0068 (0.019)	0.0051 (0.0068)	0.37
Mean (SD) Length of Stay	0.95 (1.96)	5.08 (6.05)	<0.001

*Other was based on patient preference as entered by the nurse in the electronic medical record.

© 2024 Banerjee J et al. *JAMA Network Open.*

eTable 8. Characteristics of Patients With Osteomyelitis

Characteristic	Safer@Home Cohort	Control Cohort	P Value
Care Setting at Discharge--no.(%)			
Hospital	4 (23.5%)	32 (100%)	
Emergency Department / Observation / Urgent Care	13 (76.5%)	0 (0%)	
Total	17	32	
Mean (SD) Age (years)	52.5 (22.7)	48.4 (15.6)	0.64
Sex--no.(%)			0.80
Female	1 (25%)	10 (31.3%)	
Male	3 (75%)	22 (68.8%)	
Race/Ethnicity--no.(%)			0.74
Asian	0 (0%)	0 (0%)	
Black	0 (0%)	4 (12.5%)	
Hispanic	4 (100%)	21 (65.6%)	
White	0 (0%)	3 (9.4%)	
Other*	0 (0%)	4 (12.5%)	
Mean (SD) Case Mix Index	2.28 (1.56)	1.67 (0.68)	0.16
Mean (SD) Expected Mortality	0.0032 (0.0041)	0.0052 (0.0061)	0.54
Mean (SD) Length of Stay	0.74 (0.44)	7.91 (8.00)	<0.001

*Other was based on patient preference as entered by the nurse in the electronic medical record.

© 2024 Banerjee J et al. *JAMA Network Open.*

eTable 9. Characteristics of Patients With Pyelonephritis

Characteristic	Safer@Home Cohort	Control Cohort	P Value
Care Setting at Discharge--no.(%)			
Hospital	123 (60%)	349 (100%)	
Emergency Department / Observation / Urgent Care	82 (40%)	0 (0%)	
Total	205	349	
Mean (SD) Age (years)	53.8 (15.1)	53.8 (17.8)	0.98
Sex--no.(%)			0.39
Female	87 (70.7%)	230 (65.9%)	
Male	37 (30.1%)	119 (34.1%)	
Race/Ethnicity--no.(%)			0.95
Asian	5 (4.1%)	16 (4.6%)	
Black	4 (3.3%)	13 (3.7%)	
Hispanic	106 (86.2%)	287 (82.2%)	
White	3 (2.4%)	11 (3.2%)	
Other*	6 (4.9%)	22 (6.3%)	
Mean (SD) Case Mix Index	1.14 (0.441)	1.11 (0.407)	0.58
Mean (SD) Expected Mortality	0.0069 (0.0099)	0.0097 (0.017)	0.08
Mean (SD) Length of Stay	1.08 (0.81)	4.98 (13.45)	<0.001

*Other was based on patient preference as entered by the nurse in the electronic medical record.

© 2024 Banerjee J et al. *JAMA Network Open.*

eTable 10. Characteristics of Patients With Bacterial Pneumonia

Characteristic	Safer@Home Cohort	Control Cohort	P Value
Care Setting at Discharge--no.(%)			
Hospital	31 (53.4%)	210 (100%)	
Emergency Department / Observation / Urgent Care	27 (46.6%)	0 (0%)	
Total	58	210	
Mean (SD) Age (years)	58.8 (20.3)	52.6 (26.7)	0.21
Sex--no.(%)			0.86
Female	15 (48.4%)	98 (46.7%)	
Male	16 (51.6%)	112 (53.3%)	
Race/Ethnicity--no.(%)			0.74
Asian	3 (9.7%)	11 (5.2%)	
Black	0 (0%)	5 (2.4%)	
Hispanic	26 (83.9%)	180 (85.7%)	
White	0 (0%)	2 (1.0%)	
Other*	2 (6.5%)	12 (5.7%)	
Mean (SD) Case Mix Index	1.46 (0.39)	1.37 (0.46)	0.30
Mean (SD) Expected Mortality	0.023 (0.044)	0.024 (0.066)	0.84
Mean (SD) Length of Stay	1.68 (2.05)	4.29 (9.19)	0.03

*Other was based on patient preference as entered by the nurse in the electronic medical record.

© 2024 Banerjee J et al. *JAMA Network Open.*

eTable 11. Characteristics of Patients With COPD Exacerbation

Characteristic	Safer@Home Cohort	Control Cohort	P Value
Care Setting at Discharge--no.(%)			
Hospital	11 (52.4%)	7 (100%)	
Emergency Department / Observation / Urgent Care	10 (47.6%)	0 (0%)	
Total	21	7	
Mean (SD) Age (years)	63.4 (9.71)	60.3 (15)	0.60
Sex--no.(%)			0.78
Female	4 (36.4%)	3 (42.9%)	
Male	7 (63.6%)	4 (57.1%)	
Race/Ethnicity--no.(%)			0.70
Asian	0 (0%)	0 (0%)	
Black	3 (27.3%)	3 (42.9%)	
Hispanic	3 (27.3%)	3 (42.9%)	
White	2 (18.2%)	0 (0%)	
Other*	3 (27.3%)	1 (14.3%)	
Mean (SD) Case Mix Index	1.19 (0.47)	1.34 (0.56)	0.55
Mean (SD) Expected Mortality	0.025 (0.041)	0.026 (0.043)	0.96
Mean (SD) Length of Stay	2.03 (2.50)	9.71 (11.61)	0.03

*Other was based on patient preference as entered by the nurse in the electronic medical record.

© 2024 Banerjee J et al. *JAMA Network Open.*

eTable 12. Characteristics of Patients With CHF

Characteristic	Safer@Home Cohort	Control Cohort	P Value
Care Setting at Discharge--no.(%)			
Hospital	18 (39%)	202 (100%)	
Emergency Department / Observation / Urgent Care	28 (61%)	0 (0%)	
Total	46	202	
Mean (SD) Age (years)	67 (12)	65 (14)	0.50
Sex--no.(%)			0.69
Female	8 (44%)	80 (40%)	
Male	10 (56%)	122 (60%)	
Race/Ethnicity--no.(%)			0.95
Asian	3 (17%)	22 (11%)	
Black	2 (11%)	24 (12%)	
Hispanic	12 (67%)	143 (71%)	
White	0 (0%)	2 (1.0%)	
Other*	1 (5.6%)	11 (5.4%)	
Mean (SD) Case Mix Index	1.4 (0.5)	1.4 (0.21)	0.81
Mean (SD) Expected Mortality	0.019 (0.03)	0.027 (0.041)	0.40
Mean (SD) Length of Stay	1.81 (1.83)	5.03 (5.01)	<0.001

*Other was based on patient preference as entered by the nurse in the electronic medical record.

© 2024 Banerjee J et al. *JAMA Network Open.*

eTable 13. Characteristics of Patients With Asthma

Characteristic	Safer@Home Cohort	Control Cohort	P Value
Care Setting at Discharge--no.(%)			
Hospital	17 (71%)	104 (100%)	
Emergency Department / Observation / Urgent Care	7 (29%)	0 (0%)	
Total	24	104	
Mean (SD) Age (years)	35 (14)	36 (25)	0.98
Sex--no.(%)			0.80
Female	11 (65%)	64 (62%)	
Male	6 (35%)	40 (38%)	
Race/Ethnicity--no.(%)			0.90
Asian	1 (5.9%)	3 (2.9%)	
Black	1 (5.9%)	11 (11%)	
Hispanic	13 (76%)	77 (74%)	
White	0 (0%)	2 (1.9%)	
Other*	2 (12%)	11 (11%)	
Mean (SD) Case Mix Index	0.9 (0.16)	0.97 (0.22)	0.24
Mean (SD) Expected Mortality	0.0006 (0.00084)	0.0029 (0.015)	0.52
Mean (SD) Length of Stay	1.20 (0.81)	2.33 (2.22)	0.02

*Other was based on patient preference as entered by the nurse in the electronic medical record.

© 2024 Banerjee J et al. *JAMA Network Open.*

eTable 14. Characteristics of Patients With COVID Pneumonia

Characteristic	Safer@Home Cohort	Control Cohort	P Value
Care Setting at Discharge--no.(%)			
Hospital	11 (55%)	21 (100%)	
Emergency Department / Observation / Urgent Care	9 (45%)	0 (0%)	
Total	20	21	
Mean (SD) Age (years)	60 (27)	53 (27)	0.32
Sex--no.(%)			0.53
Female	7 (64%)	8 (38%)	
Male	4 (36%)	13 (62%)	
Race/Ethnicity--no.(%)			0.49
Asian	1 (9.1%)	1 (4.8%)	
Black	0 (0%)	4 (19%)	
Hispanic	9 (82%)	13 (62%)	
White	0 (0%)	1 (4.8%)	
Other*	1 (9.1%)	2 (9.5%)	
Mean (SD) Case Mix Index	1.7 (0.28)	1.5 (0.43)	0.19
Mean (SD) Expected Mortality	0.087 (0.16)	0.086 (0.17)	0.98
Mean (SD) Length of Stay	1.69 (1.66)	3.95 (3.64)	0.02

*Other was based on patient preference as entered by the nurse in the electronic medical record.

© 2024 Banerjee J et al. *JAMA Network Open.*

eTable 15. Characteristics of Patients With Acute Hypoxic Respiratory Failure Due to Viral infection

Characteristic	Safer@Home Cohort	Control Cohort	P Value
Care Setting at Discharge--no.(%)			
Hospital	7 (100%)	5 (100%)	
Emergency Department / Observation / Urgent Care	0 (0%)	0 (0%)	
Total	7	5	
Mean (SD) Age (years)	70 (9.3)	79 (9.7)	0.12
Sex--no.(%)			0.42
Female	4 (57%)	4 (80%)	
Male	3 (43%)	1 (20%)	
Race/Ethnicity--no.(%)			0.44
Asian	0 (0%)	1 (20%)	
Black	0 (0%)	1 (20%)	
Hispanic	6 (86%)	3 (60%)	
White	0 (0%)	0 (0%)	
Other*	1 (14%)	0 (0%)	
Mean (SD) Case Mix Index	1.4 (0.43)	1.5 (0.38)	0.51
Mean (SD) Expected Mortality	0.007 (0.0061)	0.095 (0.12)	0.08
Mean (SD) Length of Stay	1.43 (0.79)	5.20 (2.17)	0.002

*Other was based on patient preference as entered by the nurse in the electronic medical record.

© 2024 Banerjee J et al. *JAMA Network Open.*

eTable 16. Multivariable Linear Regression Model of Predictors of Observed Hospital Length of Stay (Days)

Variable	Regression Coefficient (SE)	Standardized Coefficients Beta	P-value
**Constant**	-0.12 (0.56)		0.84
**Safer@Home Cohort**	-2.82 (0.46)	-0.13	<0.001
**MS-DRG Relative Weight**	3.83 (0.46)	0.20	<0.001
**Expected Mortality**	28.87 (8.73)	0.08	<0.001

© 2024 Banerjee J et al. *JAMA Network Open.*

eTable 17. Sensitivity Analysis, Observed Lengths of Stay for Enrollment and Matched Non-Enrollment Cohorts

**Diagnosis**	**Safer@Home**		**LA General Concurrent Controls**			**LA General Baseline Controls**			**Harbor-UCLA Concurrent Controls**			**Olive View-UCLA Concurrent Controls**		
	N	Avg. LOS (SD)	N	Avg. LOS (SD)	p-value	N	Avg. LOS (SD)	p-value	N	Avg. LOS (SD)	p-value	N	Avg. LOS (SD)	p-value
Cellulitis	126	0.99 (0.97)	304	4.36 (7.49)	<0.001	413	4.37 (6.45)	<0.001	232	4.11 (8.24)	<0.001	124	3.5 (3.53)	0.001
Diabetic Foot Infection	299	0.92 (1.6)	120	5.08 (6.05)	<0.001	219	4.89 (6.97)	<0.001	137	4.37 (4.92)	<0.001	82	3.99 (2.97)	<0.001
Osteomyelitis	17	0.78 (0.56)	32	7.91 (8.00)	0.058	64	5.86 (10.9)	0.21	31	7.77 (7.65)	0.068	13	7.31 (8.99)	0.26
Pyelonephritis/ cUTI	205	1.01 (0.86)	349	4.98 (13.4)	<0.001	595	3.79 (5.88)	<0.001	427	4.02 (6.89)	<0.001	294	4.35 (6.38)	<0.001
Bacterial Pneumonia	58	1.69 (2.13)	210	4.29 (9.19)	0.042	434	5.62 (6.67)	<0.001	242	3.84 (3.59)	0.12	162	4.12 (5.53)	0.08
COPD Exacerbation	21	2.01 (2.59)	7	9.71 (11.6	0.020	4	4.75 (2.63)	0.87	3	3.33 (2.31)	0.99	2	7.5 (2.12)	0.62
CHF	46	1.29 (1.71)	202	5.02 (5.01)	<0.001	298	4.52 (5.16)	0.003	144	5.78 (5.53)	<0.001	121	6.94 (8.85)	<0.001
Asthma/Bronchitis	24	1.13 (1.02)	104	2.33 (2.22)	0.13	99	2.26 (1.85)	0.17	60	3.07 (2.75)	0.004	31	3.29 (3.34)	0.005
COVID pneumonia	20	1.72 (1.74)	21	3.95 (3.64)	0.53	316	4.06 (5.34)	0.20	63	3.75 (4.66)	0.44	43	4.51 (3.25)	0.19
Acute Respiratory Failure Due to Viral Infection	7	1.14 (1.04)	5	5.2 (2.17)	0.018	3	3.33 (1.53)	0.49	2	3.5 (3.54)	0.56	4	2.25 (2.5)	0.88
All Diagnoses	876	1.33 (2)	1590	5.34 (10.4)	<0.001	2776	4.96 (7.75)	<0.001	1492	4.8 (7.06)	<0.001	995	5.29 (10.4)	<0.001
All Core Diagnoses	823	1.12 (1.38)	1354	4.62 (9.09)	<0.001	2445	4.43 (6.19)	<0.001	1341	4.26 (6.18)	<0.001	876	4.52 (5.98)	<0.001
All Other Diagnoses	53	4.54 (5.05)	236	9.46 (15.6)	0.25	331	8.87 (14.3)	0.35	151	9.6 (11.4)	0.27	119	10.9 (24.8)	0.11

© 2024 Banerjee J et al. *JAMA Network Open.*

eTable 18. Hospital Admission Rates From Emergency Department for the Three Most Frequently Enrolled Safer@Home Diagnoses

Facility and Diagnosis	Percent Admissions, Study Period	Percent Admissions, Baseline Period	Relative Risk (95% CI) of Admission, Study/Baseline	p-value
**LA General**				
Cellulitis	7.65% (424/5544)	8.73% (413/4733)	0.88 (0.77 - 0.99)	0.05
Diabetic Foot Infection	31.74% (186/586)	49.8% (209/420)	0.64 (0.55 - 0.74)	<0.001
Pyelonephritis	12.20% (536/4392)	14.32% (622/4343)	0.85 (0.77 - 0.95)	0.004
**Harbor-UCLA**				
Cellulitis	9.90% (248/2505)	9.59% (221/2304)	1.03 (0.87 - 1.23)	0.72
Diabetic Foot Infection	40.70% (157/386)	43.56% (142/326)	0.93 (0.79 - 1.11)	0.44
Pyelonephritis	14.42% (457/3169)	13.79% (452/3278)	1.05 (0.93 - 1.18)	0.47
**Olive View-UCLA**				
Cellulitis	7.08% (148/2090)	7.80% (143/1833)	0.908 (0.73 - 1.13)	0.39
Diabetic Foot Infection	36.80% (85/231)	44.0% (96/218)	0.84 (0.67 - 1.05)	0.12
Pyelonephritis	12.63% (325/2573)	12.65% (284/2245)	1.00 (0.86 - 1.16)	0.98

© 2024 Banerjee J et al. *JAMA Network Open.*
