# Supplementary material for: Virtual Home Care for Patients With Acute Illness
Source: JAMA Netw Open. 2024 Nov 26;7(11):e2447352. doi: 10.1001/jamanetworkopen.2024.47352 (PMC11600231; doi:10.1001/jamanetworkopen.2024.47352)
Supplement: Supplement 2. — Data Sharing Statement [file jamanetwopen-e2447352-s002.pdf]

Data Sharing Statement

Banerjee. Virtual Home Care for Patients with Acute Illness. *JAMA Netw Open*. Published November 26, 2024. doi:10.1001/jamanetworkopen.2024.47352

Data
Data available: No
